# Differential expression of villin and advillin by neuroendocrine and tuft cells in the murine lower airways

**DOI:** 10.1007/s00441-025-04003-y

**Published:** 2025-09-01

**Authors:** Wafaa Mahmoud, Alexander Perniss, Krupali Poharkar, Maryam Keshavarz, Ulrich Gärtner, Johannes Oberwinkler, Burkhard Schütz, Thomas Worzfeld, Stefan Offermanns, Wolfgang Kummer

**Affiliations:** 1 Institute for Anatomy and Cell Biology, German Center for Lung Research, Excellence Cluster Cardio-Pulmonary Institute (CPI), Justus Liebig University Giessen, 35392 Giessen, Germany; 2 Department of Anatomy, Faculty of Medicine, Jordan University of Science and Technology, Irbid, 22110 Jordan; 3 Division of Allergy and Clinical Immunology, Jeff and Penny Vinik Center for Allergic Disease Research, Brigham & Women’s Hospital and Department of Medicine, Harvard Medical School, Boston, 02115 USA; 4 Anatomy and Cell Biology, Institute of Theoretical Medicine, Faculty of Medicine, University of Augsburg, 86159 Augsburg, Germany; 5 Institute for Anatomy and Cell Biology, Justus Liebig University Giessen, 35392 Giessen, Germany; 6 Institut Für Physiologie Und Pathophysiologie, Philipps-Universität Marburg, Marburg, Germany; 7 Institute of Anatomy and Cell Biology, Philipps-University, 35037 Marburg, Germany; 8 Institute of Pharmacology, Philipps-University Marburg, 35043 Marburg, Germany; 9 Max-Planck-Institute for Heart and Lung Research, Excellence Cluster Cardio-Pulmonary Institute (CPI), 61231 Bad Nauheim, Germany

**Keywords:** Tuft cells, Brush cells, Neuroendocrine cells, Villin, Advillin

## Abstract

Previous studies identified a rare cell type in the mouse tracheal epithelium with immunoreactivity to the microvillus protein villin (*Vil1*), which persisted in mice lacking tuft cells due to deletion of the transcription factor Pou2f3. This study aimed to clarify the identity of this ill-defined cell type. Ultrastructurally, all cells with tightly packed microvilli observed in the tracheal epithelium of *Pou2f3*^−/−^-mice contained basally located dense core vesicles, a characteristic feature of neuroendocrine cells (NEC). Accordingly, immunofluorescence double-labeling utilizing NEC markers revealed villin-labeling in two thirds of NEC in the trachea, a reporter mouse strain showed Cre recombinase activity driven by the *Vil1* promoter in a subpopulation of tracheal NEC, and analysis of single cell RNA sequencing data revealed *Vil1*-mRNA expression by tracheal NEC. Notably, only a minimal fraction (≈1%) of bronchopulmonary NEC (solitary and clustered in neuroepithelial bodies) displayed villin-immunoreactivity, despite nearly half of them having a history of *Vil1* promoter activity. Microvilli of tuft cells differed ultrastructurally from those of NEC, and the majority of tuft cells were immunoreactive to advillin (*Avil*), showed *Avil* promoter activity as indicated by a reporter mouse strain, and expressed Avil-mRNA in the sequencing data set. This study uncovers villin-expressing cells in the lower airways as a cell population hidden among NEC. Advillin, not villin, is identified as a marker for airway tuft cells. This should be considered in interpreting findings based on the use of villin as a marker or Cre-driver when investigating rare cells in the murine airways.

**Graphical Abstract:**

In mice, neuroendocrine cells express villin and persist in Pou2f3-deficient mice. Tuft cells, on the other hand, express advillin and are absent in Pou2f3-deficient mice.

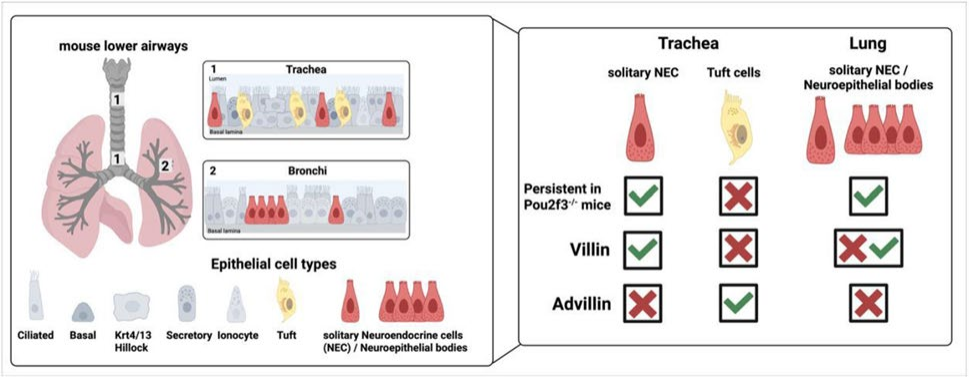

**Supplementary Information:**

The online version contains supplementary material available at 10.1007/s00441-025-04003-y.

## Introduction

The lining epithelium of the conducting airways is built up by basal, secretory, and ciliated cells as major cell types, and a variety of rare cells, each making up only around 1% of all epithelial cells. The first of them to be discovered were neuroendocrine cells (NEC) and tuft cells (TC; also termed brush or solitary cholinergic chemosensory cells), both originally defined by morphological and histochemical features. Despite being rare, these cells can play major roles in airway physiology and disease (Kotas et al., 2023; Zhu & Sun 2024). Both TC and NEC can initiate protective airway reflexes (Hollenhorst et al. 2022; Krasteva et al. 2011; Saunders et al. 2014; Seeholzer and Julius 2024; Tizzano et al. 2010), TC drive mucociliary clearance upon activation by metabolites accumulating during bacterial infection (Hollenhorst et al. 2020; Perniss et al. 2023, 2020), and both NEC and TC can give rise to various forms of small cell lung cancer (Huang et al. 2018; Park et al. 2011; Sutherland et al. 2011; Wu et al. 2022; Yamada et al. 2022).

NEC occur either solitary or clustered as neuroepithelial bodies (NEB) primarily at branching points of intrapulmonary airways. They are characterized by accumulations of secretory granules (dense core vesicles = DCV) with biogenic amines and peptides in their basal cytoplasm (Bensch et al. 1965; Frohlich 1949; Lauweryns et al. 1972; Tsutsumi et al. 1983). Commonly used markers to identify NEC are the neuroendocrine-specific ubiquitin carboxy-terminal hydrolase L1 (UCHL1), also referred to as protein gene product (PGP) 9.5 (PGP9.5), and their secretory products, e.g., calcitonin gene-related peptide (CGRP; derived from a precursor encoded by the gene *Calca* (calcitonin related polypeptide alpha) in mice (Luts et al. 1991).

TC are characterized by a brush or tuft of stiff microvilli at their apical surface, and antibodies against the microvillar actin-binding protein villin (gene name: *Vil1*) are commonly used as a marker for their visualization (Hofer and Drenckhahn 1996; Rhodin and Dalhamn 1956). Later studies on intestinal TC revealed the expression of additional members of the gelsolin/villin family, i.e., supervillin (*Svil*) and advillin (*Avil*) (Bezencon et al. 2008). Among them, advillin turned out to be characteristic for intestinal TC, and it was proposed that previous immunolabeling with villin-antibodies might have been the result of cross reactivity with advillin, which shares high sequence homology with villin (Bezencon et al. 2008; Esmaeilniakooshkghazi et al. 2020; Ruppert et al. 2020). This issue has not been clarified yet in the airways. Tracheal TC express the components of a signal transduction cascade originally identified in oropharyngeal taste cells, among them the taste-specific G protein subunit alpha transducin 3 (GNAT3; also referred to as α-gustducin) and the cation channel transient receptor potential cation channel subfamily M member 5 (TRPM5) (Kaske et al. 2007; Krasteva et al. 2011; Perniss et al. 2020). Although lacking DCV, they also produce and release mediators, among them, acetylcholine, generated by the enzyme choline acetyltransferase (ChAT), which is best characterized in airway TC’s (Krasteva et al. 2011; Perniss et al. 2020). Accordingly, GNAT3, TRPM5, and ChAT are widely accepted markers for airway TC. Their development is critically dependent on the transcription factor Pou2f3 (POU class 2 homeobox 3) (Yamashita et al. 2017).

A third rare cell type, the ionocyte, has just recently been identified by single-cell RNA sequencing (scRNA-seq) analyses (Montoro et al. 2018; Plasschaert et al. 2018). It is characterized by high expression of the cystic fibrosis transmembrane conductance regulator (CFTR) and further ion transporters, and a role in the pathogenesis of cystic fibrosis has been proposed (Yuan et al. 2023).

In addition to these established cell populations, findings obtained when studying TC utilizing villin-antibodies as a marker pointed toward the existence of a fourth, yet ill-characterized rare cell type in the mouse trachea. It was noted early that not all villin-immunoreactive solitary cells of the tracheal epithelium express ChAT (Krasteva et al. 2011), and further studies employing two independent genetic models validated the occurrence of villin-positive non-TCs. Tracheas from *Pou2f3* gene-deficient mice and animals expressing diphtheria toxin A driven by the *Trpm5* promoter completely lack cells expressing canonical TC markers (TRPM5, GNAT3, ChAT) but still harbor villin-positive cells in their epithelium (Perniss et al. 2020; Yamashita et al. 2017). We here set out to determine the identity of this ill-defined villin-positive cell in the murine airway epithelium by analyzing villin expression at mRNA and protein level and epithelial ultrastructure in wild-type mice and appropriate knockout and reporter mouse strains. In view of the high sequence homology between villin and advillin and the resulting confusion concerning intestinal TC, we analyzed advillin expression in parallel.

## Materials and methods

### Animals

Mice were housed under specified pathogen-free (SPF) conditions (10 h dark, 14 h light) with free access to food and water. This study was carried out in accordance with the recommendations of the European Communities Council Directive of 24th November 1986 (86/609/EEC). The protocol was approved by the local authorities. Mice at least 12 weeks of age and from both genders were used. Mice were killed by inhalation of an overdose of 5% isoflurane (Abbott, Wiesbaden, Germany) and exsanguination through abdominal blood vessels. The following mouse strains were used (for further details see Supplementary Tab. 1): Wild-type C57BL/6Rj mice (Janvier Labs, Le Genest-Saint-Isle, France, Cat#5,751,862) (*n* = 20), *Chat-*eGFP (enhanced green fluorescent protein) (B6.Cg-Tg(RP23-268L19-EGFP)2Mik/J, Jackson Laboratory, Bar Harbour, USA, Cat# JAX: 007902) (*n* = 4), Pou2f3^tm1Abek^ (*Pou2f3*^−/−^) mice (*n* = 8), (Matsumoto et al. 2011). The following mouse lines were used to create *the Vil1-Cre*^+^*/mTmG*^+^ (membrane-targeted tandem Tomato/membrane-targeted green fluorescent protein) mouse strain. A double-fluorescent reporter mouse line *ROSA*^*mT/mG*^, B6.129(Cg)-Gt(ROSA)26Sortm4(ACTB − tdTomato, − EGFP)Luo/J, (MGI:3,716,464, JAX: 007676) (Muzumdar et al. 2007), and a mouse line that expresses Cre-recombinase under the control of the *Vil1* promoter (*Vil1-Cre*, B6.Cg-Tg(*Vil1-cre*)997Gum/J, (MGI:2,448,639, JAX: 004586) (Madison et al. 2002). Hemizygous *Vil1-cre* mice were bred with homozygous *ROSA*^*mT/mG*^ mice. From these crossings, the following offsprings were used: *Vil1-Cre*^+^*/mTmG*^+^ (*n* = 14), *Vil1-Cre*^*−*^*/mTmG*^+^ (*n* = 9), *Vil1-Cre*^*−*^*/mTmG*^*−*^ (*n* = 4), *Vil1-Cre*^+^*/mTmG*^*−*^ (*n* = 3). To create the *Avil1-Cre*^+^*/mTmG*^+^ mouse strain, the *Avil-Cre* line (B6;D2-Tg(*Avil*-cre)1Phep/Cnrm, European Mouse Mutant Archive repository, Id EM:05542; Infrafrontier GmbH, Neuherberg, Germany) was crossed with the same *ROSA*^*mT/mG*^ strain used for the *Vil1-Cre*^+^*/mTmG*^+^ breeding (Zurborg et al. 2011). Hemizygous *Avil-Cre* mice were bred with homozygous *ROSA*^*mT/mG*^ mice, and the offspring were analyzed (*n* = 3) (Ruppert et al. 2020).

### 3R statement

In this study, more than 150 tissue specimens were collected, investigated, and analyzed. In order to adhere to the 3R principle (reduction, replacement, and refinement) in animal experiments principle (Russell 1995), the number of animals used for this study was kept to a minimum by taking multiple organs (urethra, trachea, thymus, gall bladder, thyroid gland, intestine, kidney, tongue, spleen and lung) from the same animal to be used in other projects and by taking specimens from animals that have been sacrificed for other purposes.

### Tissue preparation

Tracheas were dissected for cryosections, paraffin sections, RT-PCR (reverse transcription polymerase chain reaction), transmission, or scanning electron microscopy. For RT-PCR of the tracheal epithelium and scanning electron microscopy, the trachea was removed and opened by cutting the trachealis muscle longitudinally to expose the lining epithelium. The trachea was then pinned on a piece of wax to align the epithelial layer approximately in the same plane. The trachea, lung, small intestine, and colon processed for cryosectioning were dissected either freshly or after transcardiac perfusion with Zamboni solution following an initial perfusion with a rinsing solution containing heparin (2 ml/l; 10,000 U; Ratiopharm, Ulm, Germany) and procaine hydrochloride (5 g/l; Merck), pH 7.4. Subsequently, all tissues were immersed in Zamboni’s fixative overnight at 4 °C, followed by washing steps in 0.1 M phosphate buffer (5 × 60 min) and overnight incubation for cryoprotection in 18% sucrose (Merck) in 0.1 M phosphate buffer at 4 °C. For cryopreservation, specimens were embedded in OCT cryostat sectioning medium (Sakura Finetek, Staufen, Germany), frozen in 2-methylbutane (Carl Roth) chilled by liquid nitrogen, and stored at − 20 °C until further processing. For paraffin embedding, samples were dehydrated and embedded in paraffin (Sakura Finetek). Whole tracheas and lungs processed for RT-PCR were dissected freshly and followed the protocol as described later.

### Indirect immunohistochemistry of cryosections and paraffin sections

For cryosections, 10 µm thick sections were cut using a microtome (CM-1900 cryostat; Leica) and air dried for 1 h. For paraffin sections, paraffin blocks were cut with a microtome (Microm HM 325, Thermo Scientific, Schwerte, Germany), and 7 µm thick sections were mounted on silanized microscope glass slides. Cryo and paraffin sections were incubated for 1 h with blocking solution containing 10% normal horse serum, 0.5% Tween 20 (Sigma-Aldrich), and 0.1% bovine serum albumin in PBS (phosphate buffered saline). All antibodies were diluted in PBS + S (0.005 M phosphate buffer, with 0.15 M NaCl, pH 7.4). The primary antibodies (Supplementary Tab. 2) were applied in different combinations overnight at room temperature. After a washing step, the sections were incubated for 1 h at room temperature with secondary antibodies listed in Supplementary Tab. 3. Nuclei were labeled with 4′,6-diamidino-2-phenylindole (DAPI; 1 µg/ml; D9542, Sigma-Aldrich). Afterward, samples were post-fixed in buffered 4% paraformaldehyde, washed in PBS, and coverslipped with Mowiol 4–88 (pH 8.6; Merck) or carbonate-buffered glycerol (pH 8.6, Sigma-Aldrich).

Antigen retrieval by microwave treatment for 10 min in citrate buffer (pH 6.0) was applied only before using the antibody against rabbit villin (Invitrogen, Carlsbad, California, USA, MA5-16,408). Samples were evaluated by epifluorescence (Axioplan 2, Zeiss, Oberkochen, Germany) or by confocal laser scanning microscopes (LSM 710, Zeiss; LSM 980-Airyscan 2; Zeiss) equipped with the appropriate filter sets and lasers, respectively.

### RT-PCR

Samples from whole trachea (*n* = 2) and lung (*n* = 3) from C57BL/6Rj mice were freshly dissected and soaked in RLT buffer (RNA lysis buffer, Qiagen, Hilden, Germany) supplemented with 1% β-mercaptoethanol (Sigma-Aldrich). For selective investigation of the tracheal epithelium (TE, *n* = 2 C57BL/6Rj mice), the trachea was opened, and the epithelial layer was abraded using cotton swabs soaked with RLT buffer supplemented with 1% β-mercaptoethanol and purified with a QIA shredder column (Qiagen). Total RNA from all samples was isolated by using the RNeasy kit (Qiagen) according to the manufacturer’s instructions. RNAs were stored at − 80 °C until further use.

For cDNA synthesis (Eppendorf Master Cycler Personal 5332, Hamburg, Germany), 8 µl RNA was incubated with 1 µl 10 × DNase reaction buffer and 1 µl DNase (1 U/µl; Invitrogen) for 15 min at 25 °C to degrade contaminating DNA. To each sample, 1 µl of EDTA (ethylenediaminetetraacetic acid, 25 mM) was added. After 10 min incubation at 65 °C, samples were rapidly cooled for 2 min, and 9 µl reaction mixture of (1 µl oligo-dT (50 µM), 1 µl dNTPs (10 mM), 1 µl Superscript RNase (200 U/µl), 4 µl 5 × first-strand buffer, 2 µl dithiothreitol (0.1 M)) was added to each sample. All reagents were from Invitrogen, except dNTPs which were from Qiagen. cDNAs were stored until further use at − 20 °C.

PCR was performed with cDNA samples using primers (specified below) for *Villin 1*, *Calca*, and *β-actin* (housekeeping gene). The following protocol was used: 1 µl cDNA as template, 2 µl MgCl_2_ (25 mM), 2.5 µl 10 × PCR buffer II, 0.75 µl dNTPs (10 mM), 0.75 µl of each primer (20 pM), 0.25 µl AmpliTaq Gold DNA polymerase (5 U/µl) (all reagents were purchased from Thermo Fisher), and 17.75 µl H_2_O. PCR was conducted with the following temperature and time profile: 95 °C for 12 min for initial denaturation, followed by 39 cycles at 95 °C for 20 s, 60 °C for 20 s, and 72 °C for 20 s and a final extension at 72 °C for 7 min. *β-actin* was used as an efficacy control for PCR, and omission of the reverse transcriptase during cDNA synthesis served as a negative control. Water negative controls were also processed with each reaction to check for the absence of genomic DNA contamination. Primer pairs used *β-actin* fwd: GTGGGAATGGGTCAGAAGG, rev: GGCATACAGGGACAGCACA, product length 300 bp, GeneBank accession number NM007393; *Vil1* fwd: TGGAAACCGAGACCTTGAGA, rev: TCCACTTTCGGGCTCATAAC, product length 195 bp, NM009509.2; *Calca* fwd: ATGCAGATGAAAGCCAGGGA, rev: AAGTTGTCCTTCACCACACC, product length 158 bp, NM001289444. The PCR products were subjected to electrophoresis in ethidium bromide-containing 2% agarose gels. A 100-bp DNA ladder (Invitrogen) was run as a marker, and bands were detected by UV light.

### In silico analysis of published mRNA sequencing data

Previously published (Plasschaert et al. 2018) gene expression data (GSE102580) of murine tracheal epithelial cells from uninjured C57Bl6/J mice were downloaded from SPRING and re-analyzed. Analysis and re-clustering were done using the Seurat R package (version 2.3.4) (Wolock et al. 2019). Principal component analysis (PCA) was done, and UMAP (uniform manifold approximation and projection) was used for non-linear dimensional reduction (Satija et al. 2015). Cells were represented in a two-dimensional UMAP plot, and clusters were identified and annotated based on the composition of typical marker genes.

### Electron microscopy

For transmission electron microscopy, tracheas were fixed for at least 24 h in 2% paraformaldehyde and 1.5% glutaraldehyde (Merck) in 0.1 M phosphate buffer (pH 7.4). After fixation, specimens were washed in HEPES (4-(2-hydroxyethyl)−1-piperazineethanesulfonic acid) buffer 0.15 M, pH 7.4, osmicated for 2 h in aqueous 1% osmium tetroxide (Sigma-Aldrich), washed in distilled water, contrasted in 1% uranyl acetate (Merck) overnight, washed in distilled water, and dehydrated in an ascending series of ethanol. Samples were then transferred into propylene oxide (Merck) and processed through mixtures of epon (Agar Scientific, Essex, UK) and propylene oxide (100% ethanol + propylene oxide 1:1, 15 min; pure propylene oxide 2 × 5 min; propylene oxide + epon 1:1, 60 min) before being infiltrated with epon overnight at room temperature. Specimens were then embedded in epon at 60 °C overnight, and ultrathin Sects. (80 nm) were cut using an ultramicrotome (Reichert Ultracut E, Leica). Ultrathin sections were analyzed using a transmission electron microscope (EM 902 N, Zeiss, Oberkochen, Germany) equipped with a slow scan 2 K CCD camera (TRS, Tröndle, Moorenweis, Germany). For analysis of microvilli length, width, and distance, images were captured with 12,000 magnification. For measurement of mitochondrial dimensions, images were captured with 20,000 magnification. Using ImageJ software, the two central perpendicular axes of mitochondria were measured. For measurements of apical cell width, images were captured with 7000 magnification. The evaluated ciliated and secretory cells were chosen randomly, but all showed their nucleus and contacted the basement membrane. Measurements were done using Fiji ImageJ software.

For scanning electron microscopy, tracheas from C57BL/6Rj mice (*n* = 3) were fixed for at least 24 h in 2% paraformaldehyde and 1.5% glutaraldehyde (Merck) in 0.1 M phosphate buffer (pH 7.4). After fixation, specimens were washed in HEPES buffer 0.15 M, pH 7.4, osmicated for 2 h in aqueous 1% osmium tetroxide (Sigma-Aldrich), and washed in distilled water. They were then dehydrated in an ascending series of ethanol. Samples were critical point dried by CO_2_ treatment (CPD 030 critical point dryer, Bal-Tec, Pfaffikon, Switzerland) and sputtered with gold particles (Polaron E500, UK). Samples were visualized via a Philips XL30 scanning electron microscope. For microvilli length and width measurements, images were captured with 50,000 magnification and measured using Fiji ImageJ software.

### Statistical analysis

Data sets with a large sample size (> 30 values) were tested for normality of distribution using the Kolmogorov–Smirnov and Shapiro–Wilk tests, and all were found not to be normally distributed. Accordingly, data are presented as individual data points with median ± interquartile range. Also, only non-parametric tests (Mann–Whitney U-test or Kruskal–Wallis test followed by Dunn’s multiple comparisons test) were used. Differences were considered statistically significant when *p* ≤ 0.05. Statistical analyses of data were performed with GraphPad Prism software version 7 (La Jolla, CA, USA).

## Results

### NEC are the only microvillous epithelial cell type in the trachea of Pou2f3^−/−^ mice

**Fig. 1:**
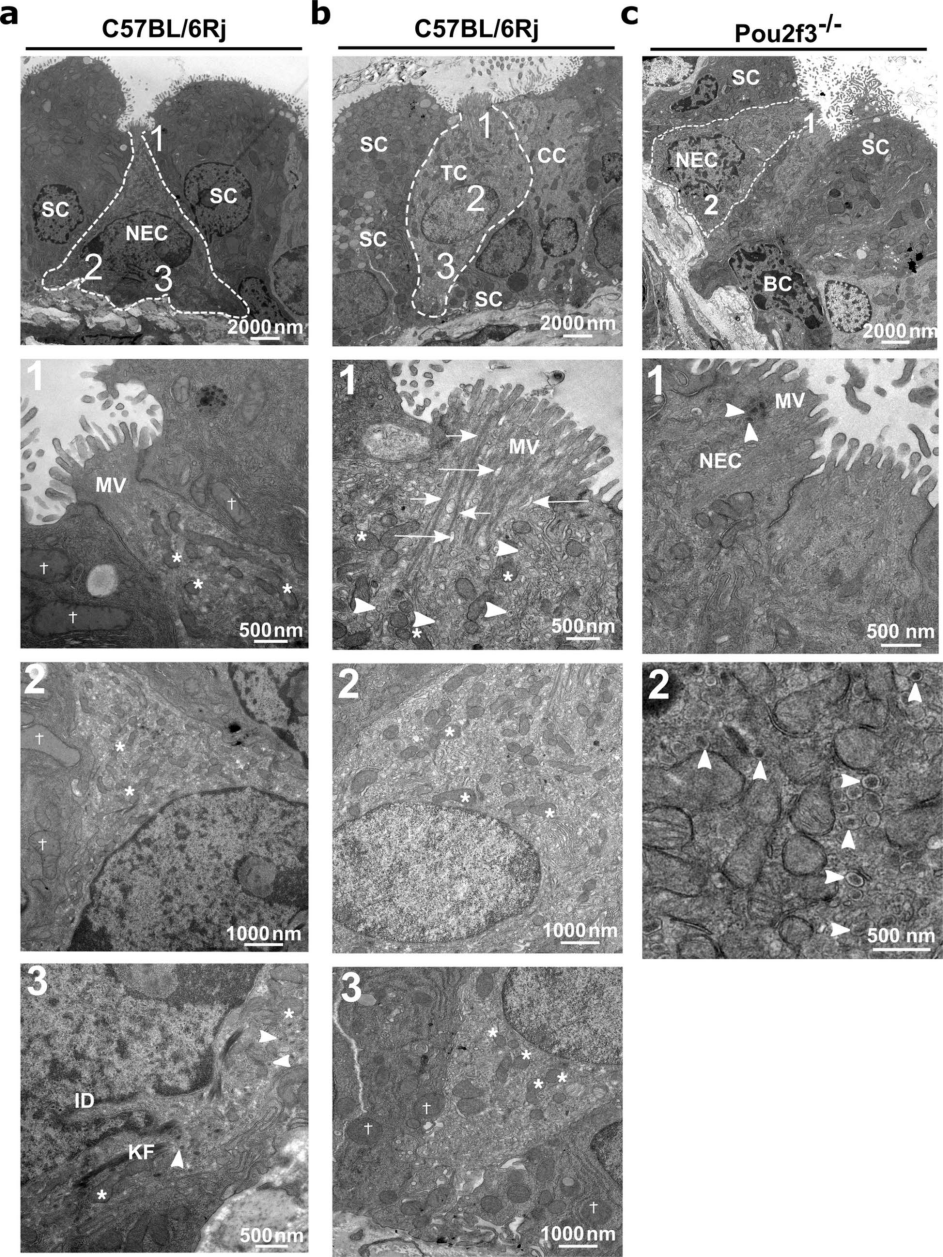
Cell types of the tracheal epithelium of wild-type C57BL/6Rj and Pou2f3^*−/−*^ mice, analyzed by TEM. **a** NEC of a C57BL/6Rj mouse. NEC is pyramidal in shape and has an irregular basal nucleus. 1 is a higher magnification of the apical region of the NEC, showing short, thick, and spaced MV. Mitochondria of the NEC (asterisks) are smaller than those of the neighboring secretory cell (crosses); see also panel 2, which represents a higher magnification of the middle region of the NEC. 3 is a higher magnification of the basal region of the NEC, showing small mitochondria (asterisks), numerous DCV (arrowheads) with variable densities and keratin filaments (KF). The nucleus is irregular with an indentation (ID). **b** Tracheal epithelium of a C57BL/6Rj mouse, TC has a flask shape and an oval to round nucleus. 1 is a higher magnification of the apical region of the TC, showing a tuft of long, tightly packed MV protruding from its small apical part. Bundles of microfilaments (short arrows) extend from the root of the MV into the cytoplasm. Few DCV (arrowheads) are visible in the apical region. Clear tubulo-vesicular membranous structures are found in the apical region (long arrows). Mitochondria are indicated by asterisks. 2 is a higher magnification of the middle region of the TC showing its small mitochondria (asterisks). 3 is a higher magnification of the basal region of TC showing its small mitochondria (asterisks) and larger mitochondria in the neighboring secretory cells (crosses). **c** Tracheal epithelium of a *Pou2f3*^*−/−*^ mouse, including SC, BC, and a NEC. 1 is a higher magnification of the apical region of the NEC, showing MV and DCV (arrowheads). 2 is a higher magnification of the basal region of the NEC, showing numerous DCV (arrowheads). BC, basal cell; CC, ciliated cell; DCV, dense core vesicles; KF, keratin filament; MV, microvilli; NEC, neuroendocrine cell; SC, secretory cell; TC, tuft cell

To investigate the ultrastructure of the poorly defined villin-immunoreactive cell in the trachea, rare microvillous epithelial cell types in wild-type C57BL/6Rj mice (*n* = 7 animals) were compared with those of *Pou2f3*^*−/−*^ mice (*n* = 5). This approach was chosen on the rationale that NEC can be identified by the presence of basally accumulating DCV, and TC are absent in *Pou2f3*^*−/−*^ but present in *Pou2f3*^±^ and C57BL/6Rj mice (Yamashita et al. 2017). Hence, in *Pou2f3*^*−/−*^ mice, rare microvillous cells without basal accumulation of DCV should represent this ill-defined villin^+^ cell type. However, while typical TC without DCV were seen in addition to NEC in C57BL/6Rj mice (Fig. 1a, b, Supplementary Fig. 1), cells with NEC features were identified as the only microvillous epithelial cell type present in *Pou2f3*^*−/−*^ mice (Fig. 1c).

### Microvilli of NEC are structurally distinct from TC microvilli

In transmission electron microscopy, NEC and TC appeared different. The most distinctive feature of the NEC was the presence of numerous DCV with an average size of 110 nm in diameter, which were concentrated in the basal region (Fig. 1a, c, Supplementary Fig. 1). NEC of *Pou2f3*^*−/−*^ and C57BL/6Rj mice were pyramidal or flask-shaped cells. Their nuclei were irregular, sometimes with indentation, and located basally (*n* = 16 cells from five *Pou2f3*^*−/−*^ mice and *n* = 5 cells from three C57BL/6Rj mice) (Fig. 1a, c). TC were found only in C57BL/6Rj mice and were characterized by apical microvilli, but no DCV accumulations; they were flask-shaped with oval or rounded nuclei (*n* = 11 cells from seven C57BL/6Rj mice) (Fig. 1b).

**Fig. 2:**
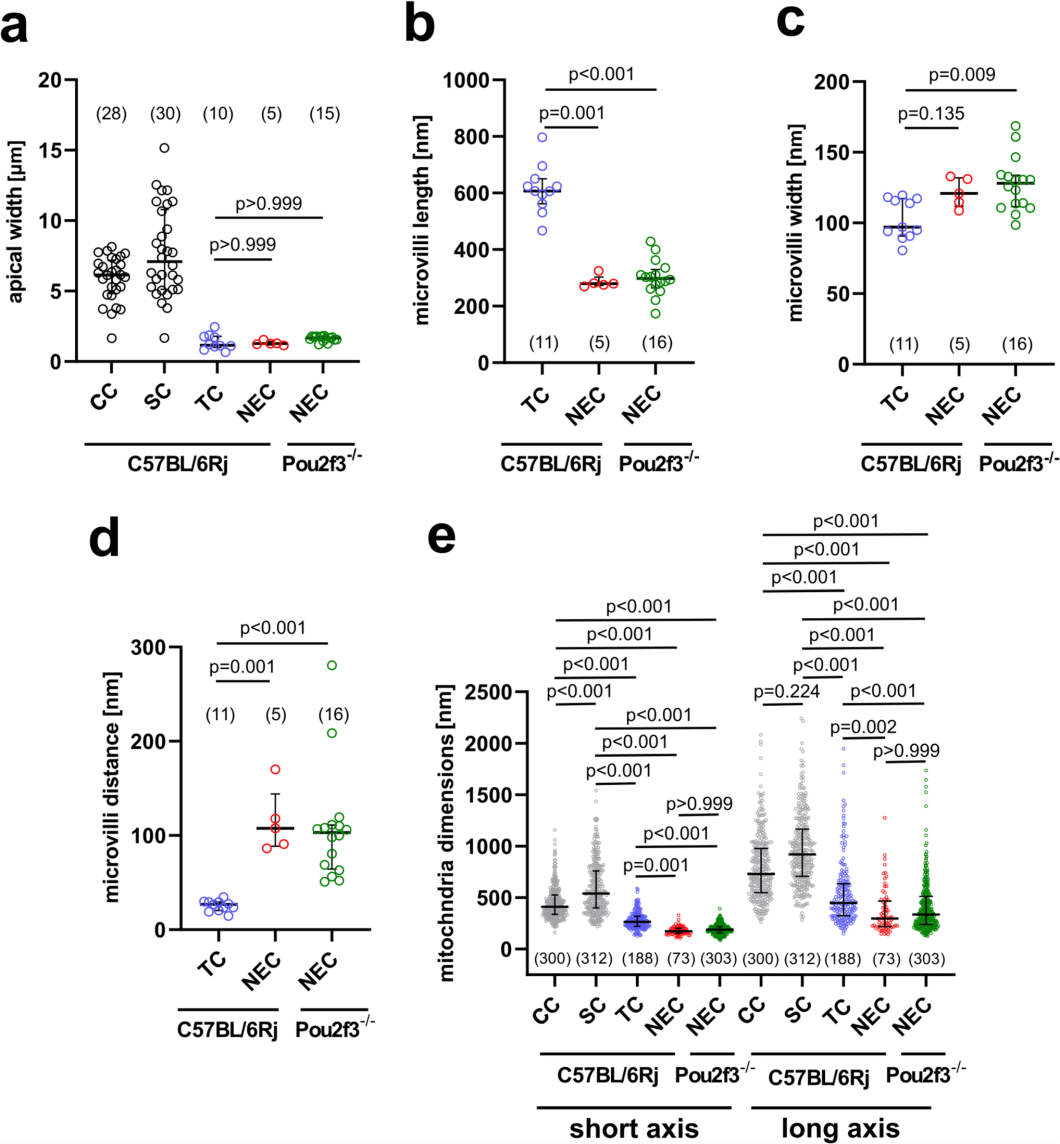
Ultrastructural dimensions of tracheal epithelial cell types determined by TEM. **a** Apical cell width, each dot represents one cell. **b** Microvilli length of TC and NEC. **c** Microvilli width of TC and NEC. **d** Distance between adjacent microvilli in TC and NEC. **e** Mitochondrial dimensions (short axis) and long axis measurements in TC, NEC, ciliated, and secretory cells. Each dot represents the measurement of mitochondrial diameter (short axis) or long axis of one mitochondrion; the data were pooled from 11 TC, 5 NEC, 28 ciliated cells, and 30 secretory cells of C57BL/6Rj mice, and 15 NEC of *Pou2f3*^*−/*^* mice*. **a**–**e** Scatter dot plots with median and interquartile range bars. **a**–**d** Each dot represents the average of one cell. **a**–**e** Kruskal–Wallis test and Dunn’s multiple comparisons test. NEC, neuroendocrine cell; SC, secretory cell; CC, ciliated cell; TC, tuft cell

The apical cell width of both NEC and TC was small, being not significantly different from each other and reaching only about 20% of that of ciliated and secretory cells (Fig. 2a, Supplementary Tab. 4). In NEC, the apical microvilli were shorter (about 50%), thicker, and less densely spaced than in TC (Fig. 2b–d, Supplementary Tab. 4). NEC had the smallest mitochondria among epithelial cell types, with mitochondria of ciliated and secretory cells being more than 2.5-fold larger in the short axis. Mitochondria of TC came close to the dimensions of those of NEC, but still were significantly larger (Fig. 2e, Supplementary Tab. 4). Bundles of filaments were often detected in the cytoplasm of NEC and TC. Clear tubule-vesicular membranous structures were found in the apical region of TC (Fig. 1b), whereas only in TC, microfilaments were observed extending from the root of microvilli into the cytoplasm (Fig. 1b).

**Fig. 3:**
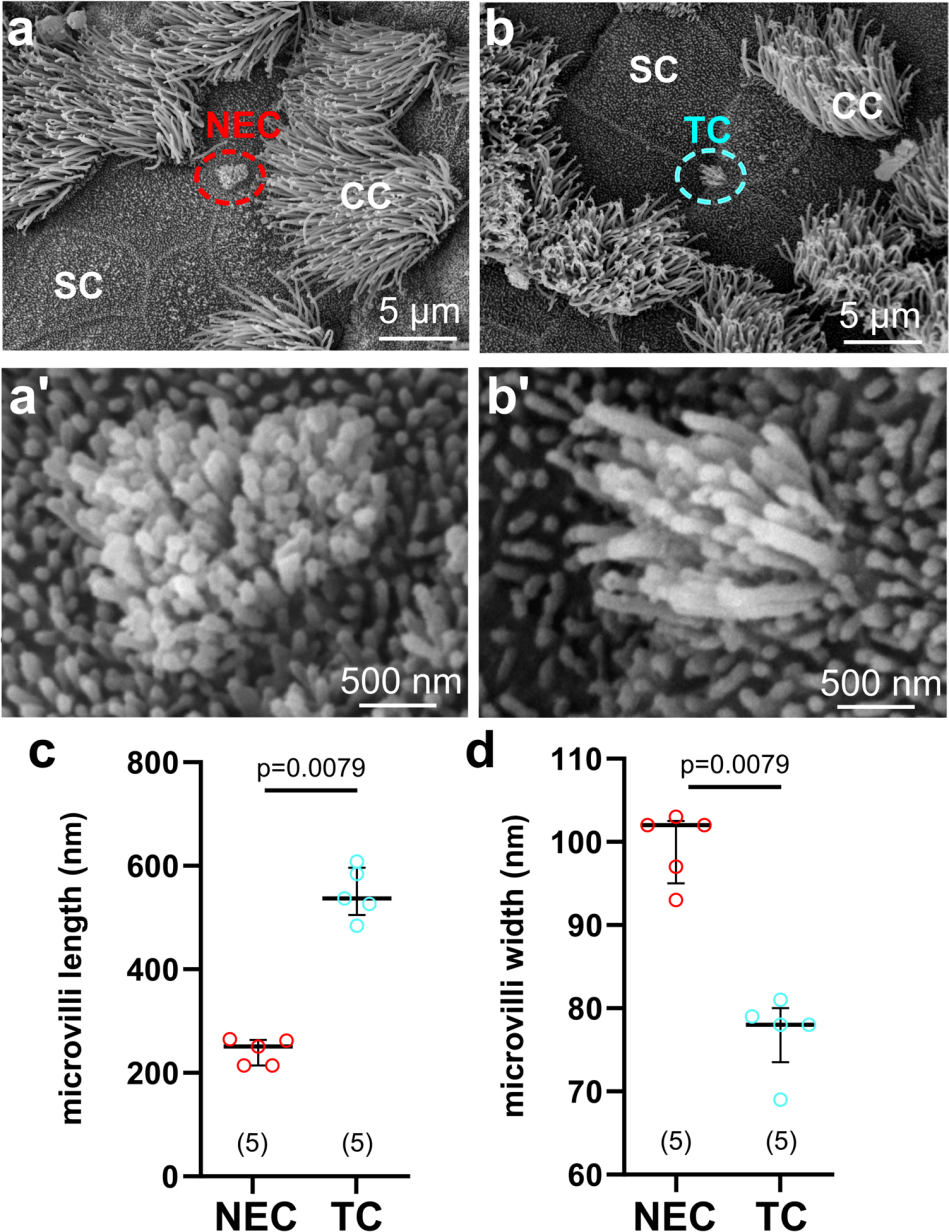
Identification of TC and NEC in a C57BL/6Rj mouse by SEM. **a** Apical part of a NEC (red circle). CC and SC are also present. (a’) Higher magnification of the NEC microvilli. The microvilli of the NEC are shorter than those of the TC. **b** Apical part of a TC (light blue circle). CC and SC are also present. (b’) Higher magnification of the TC microvilli. **c**, **d** Scatter dot plots with median and interquartile range bars. **c** Microvilli length of TC and NEC. **d** Microvilli width in TC and NEC. **c**, **d** Each dot represents the average microvillus length and width of each cell. Mann–Whitney *U* test. SC, secretory cell; CC, ciliated cell; NEC, neuroendocrine cell; TC, tuft cell

In scanning electron microscopy, ciliated cells were identified by their large luminal surface with cilia and long microvilli. Secretory cells were identified by their large surface with scattered short microvilli; sometimes, their apical surface showed a dome-like appearance (Fig. 3a, b). NEC and TC were rare and had a small apical surface covered by microvilli (Fig. 3a’, b’). Measurements of microvillus length and thickness revealed two non-overlapping populations of cells. Referring to information obtained before by transmission electron microscopy, we designated the population with short and thick microvilli in scanning electron microscopy as NEC, and those cells with long and thin microvilli as TC (Fig. 3a–d, Supplementary Tab 5).

### Villin-immunoreactive cells are positive for NEC-specific markers

**Fig. 4:**
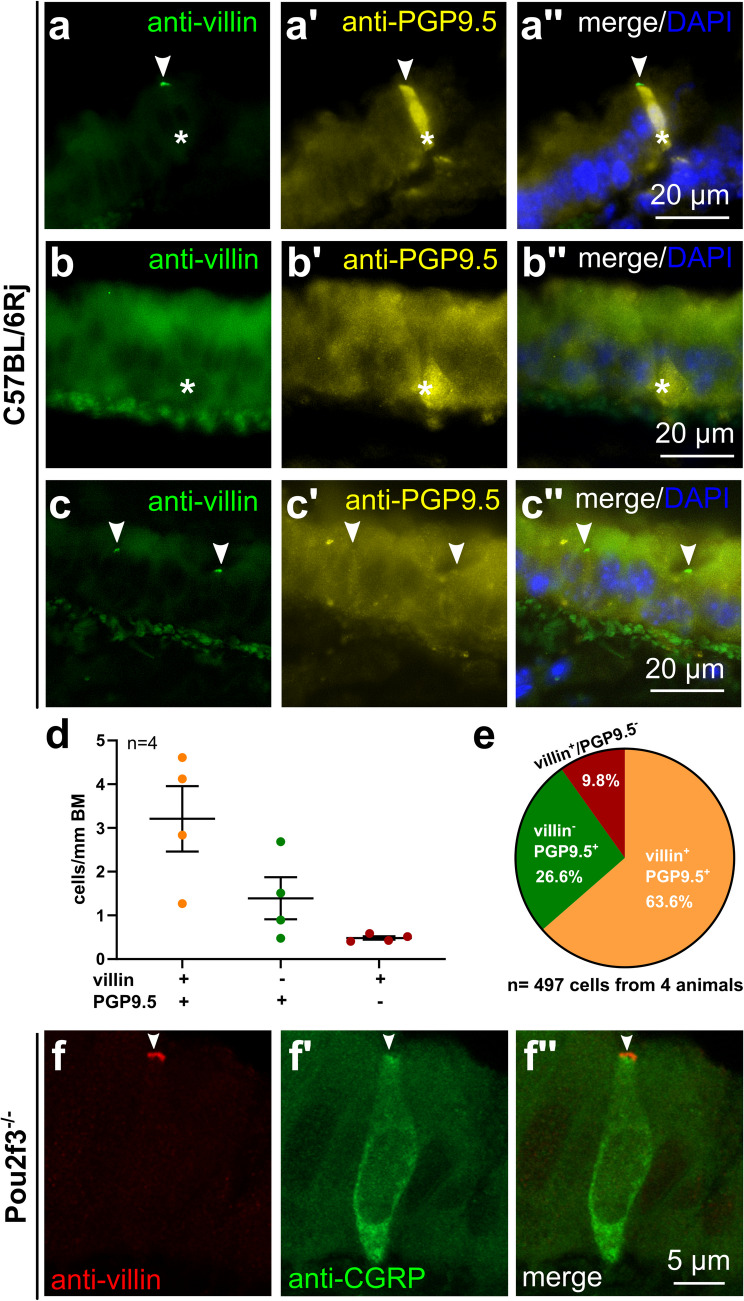
Villin- and PGP9.5-immunoreactivity in tracheal epithelium. **a**–**c** Immunohistochemistry of tracheal cryosections of C57BL/6RJ mice, immunolabeled with antibodies against villin (rabbit, monoclonal, RRID AB_2537927) and PGP9.5 (chicken, polyclonal, RRID AB_877619) (**a**)shows an epithelial cell double-positive for villin (arrowhead, tip) and PGP9.5 (asterisk, whole cell). **b** Cell only positive for PGP9.5 (asterisk), but not for villin. **c** Cells only positive for villin (arrowheads), but not for PGP9.5. **d**, **e** Tracheal cryosections from wild-type mice (C57BL/6RJ) were double-labeled with antibodies against PGP9.5 and villin. **d** Quantification of immunoreactive cells in tracheal cryosections (10 coronal sections/trachea, *n* = 4 tracheas), showing the number of villin^+^/PGP9.5^+^, villin^−^/PGP9.5^+^, and villin^+^/PGP9.5^−^ cells/mm of basement membrane (BM). **e** Pie chart showing the percentages of phenotypes illustrated in the left panel. **f** Confocal laser scanning microscopy, double-labeling immunohistochemistry of a tracheal cryosection of a *Pou2f3*^*−/−*^ animal. Epithelial cell double-positive for villin (rabbit, polyclonal, RRID AB_1968408, arrowhead) and CGRP (goat, polyclonal, RRID AB2243858). Maximum intensity projection of a *z*-stack of confocal optical sections

Based on the electron microscopic observation that all rare microvillous cells in tracheas of *Pou2f3*^*−/−*^ mice exhibited NEC features, tissue sections from C57BL/6RJ and *Pou2f3*^*−/−*^ mice were double-immunolabeled with antibodies against villin and canonical NEC-specific markers (PGP9.5 or CGRP). Different villin antibodies were used in the experiments, which resulted in a similar outcome of co-labeling of villin and NEC markers (Fig. 4 and Supplementary Fig. 2). Omission of the primary antibodies validated the specificity of the used secondary antibodies, indicated by the absence of labeling of epithelial cells (Supplementary Fig. 3). The colon was used as a positive control for all villin antibodies used in the experiments, showing labeling of the brush border of colonocytes (Supplementary Fig. 3b and d).

In the trachea, rare, flask-shaped epithelial cells exhibited villin-immunoreactivity in their apical region (Fig. 4 and Supplementary Fig. 2). Double-labeling revealed extensive colocalization with NEC markers (villin^+^/PGP9.5^+^; villin^+^/CGRP^+^), although single-positive cells (villin^−^/PGP9.5^+^, villin^+^/PGP9.5^−^, villin^−^/CGRP^+^, villin^+^/CGRP^−^) also occurred (Fig. 4a–e and Supplementary Fig. 2). Proportions of phenotypes were quantified for the antigen pair villin/PGP9.5. This approach defined two phenotypes of tracheal NEC (PGP9.5^+^) cells: Two-thirds of NEC were villin-immunoreactive, whereas one-third was not. An additional 10% of immunoreactive cells were single positive for villin (Fig. 4d–e). In the lungs, in contrast, only a minimal fraction (< 1%) of CGRP^+^ solitary broncho-pulmonary NEC and CGRP^+^ cells of NEB was villin-immunoreactive (Supplementary Fig. 4).

**Fig. 5:**
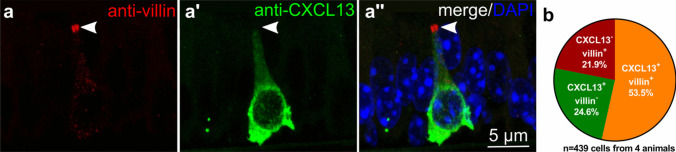
Villin- and CXCL13-immunoreactivity in the tracheal epithelium. **a** Confocal laser scanning microscopy, maximum intensity projection of a *z*-stack of confocal optical sections. Tracheal cryosection of a C57BL/6RJ mouse, immunolabeled with antibodies against villin (rabbit, monoclonal, RRID AB_2537927) and CXCL13 shows an epithelial cell double-positive for villin (arrowhead, tip) and CXCL13. **b** Pie chart showing the percentage of villin^+^/CXCL13^+^, villin^−^/CXCL13^+^, and villin^+^/CXCL13.^−^ cells related to the pooled total number of immunoreactive cells (10 coronal sections/trachea, *n* = 4 tracheas)

We previously identified a high homeostatic expression level of CXCL13 (C-X-C motif chemokine ligand 13) in a subpopulation of tracheal NEC (Mahmoud et al. 2022). Double-labeling of tracheal cryosections from wild-type mice (C57BL/6RJ, *n* = 4) showed co-labeling of villin and CXCL13 in more than half of the immunoreactive cells, while single-positive cells (villin^+^/CXCL13^−^, villin^−^/CXCL13^+^) each accounted for about a quarter (Fig. 5).

**Fig. 6:**
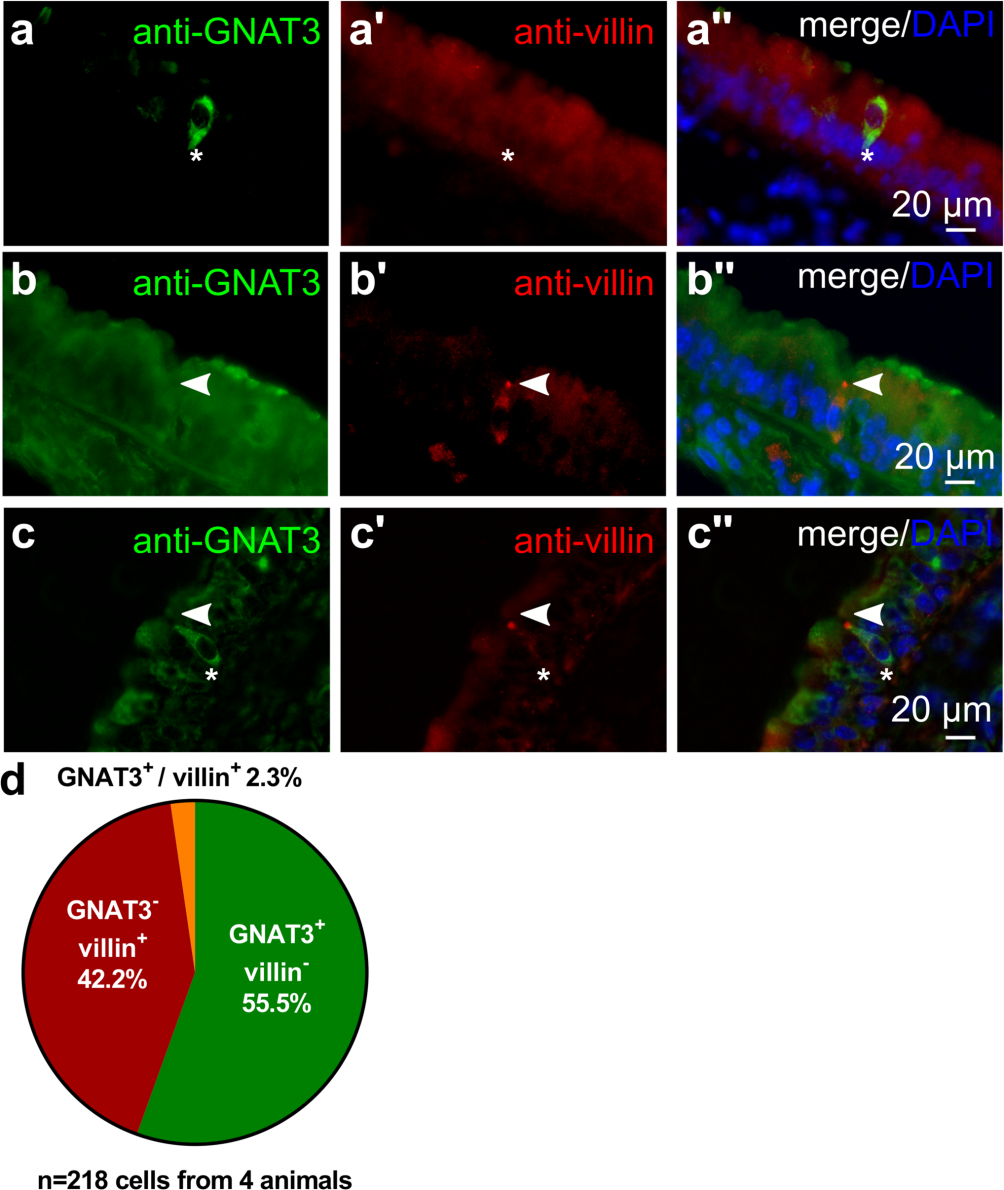
Villin- and GNAT3-immunoreactivity in the tracheal epithelium. **a**–**c** Tracheal cryosections of C57BL/6RJ mice, immunolabeled with antibodies against villin (rabbit, monoclonal, RRID AB_2537927) and GNAT3. **a** Cell only positive for GNAT3 (asterisk) but not for villin. **b** Cell only positive for villin (arrowhead) but not for GNAT3. **c** An epithelial cell, double-positive for villin (arrowhead, tip) and GNAT3 (asterisk, whole cell). **d** Pie chart showing the percentage of villin^+^/GNAT3^+^, villin^−^/GNAT3^+^, and villin^+^/GNAT3.^−^ cells related to the pooled total number of immunoreactive cells (6–8 coronal sections/trachea, *n* = 4 tracheas)

Immunolabeling of tracheal sections with antibodies against the TC marker GNAT3 and villin revealed that TC were only rarely villin^+^/GNAT3^+^ (2.3%) (Fig. 6).

### A subpopulation of NEC expresses Vil1-Cre activity

To further validate the expression of villin in a subpopulation of NEC, mice expressing Cre-recombinase driven by the villin promoter (*Vil1-Cre*^+^) were crossed with mice carrying *loxP* sites on either side of a membrane-targeted tdTomato (tandem-dimer Tomato, mT) cassette followed by a membrane-targeted eGFP (mG) cassette (ROSA^mT/mG^). In *Vil1-Cre*^+^*/mTmG*^+^ offspring, cells with *Vil1*-driven Cre-recombinase activity display GFP, but no tdTomato fluorescence, whereas all other cells show tdTomato but no GFP fluorescence.

The small intestine served as a positive control. Native eGFP fluorescence was detected in the whole intestinal epithelium in *Vil1-Cre*^+^*/mTmG*^+^ animals (Supplementary Fig. 5a-b). When sections were immunolabeled with an antibody against eGFP, all native eGFP^+^ cells were immunoreactive to eGFP antibody, and no additional immunoreactive cells were visible (Supplementary Fig. 5a, b). Double-immunolabeling with antibodies against eGFP and villin revealed that Cre-recombinase had been active only in villin-immunoreactive intestinal epithelial cells (Supplementary Fig. 5c). Red tdTomato fluorescence was present in every cell which was not green in *Vil1-Cre*^+^*/mTmG*^+^ animals (Supplementary Fig. 5a, b). As expected, all cells showed red fluorescence in *Vil1-Cre*^*−*^*/mTmG*^+^ animals, but no eGFP signals, neither native nor with eGFP-immunolabeling (Supplementary Fig. 5 d). In *Vil1-Cre*^*−*^*/mTmG*^*−*^ and in *Vil1-Cre*^+^*/mTmG*^*−*^mice, neither red tdTomato fluorescence nor native or antibody-enhanced eGFP signals were detected (Supplementary Fig. 5e, f).

**Fig. 7:**
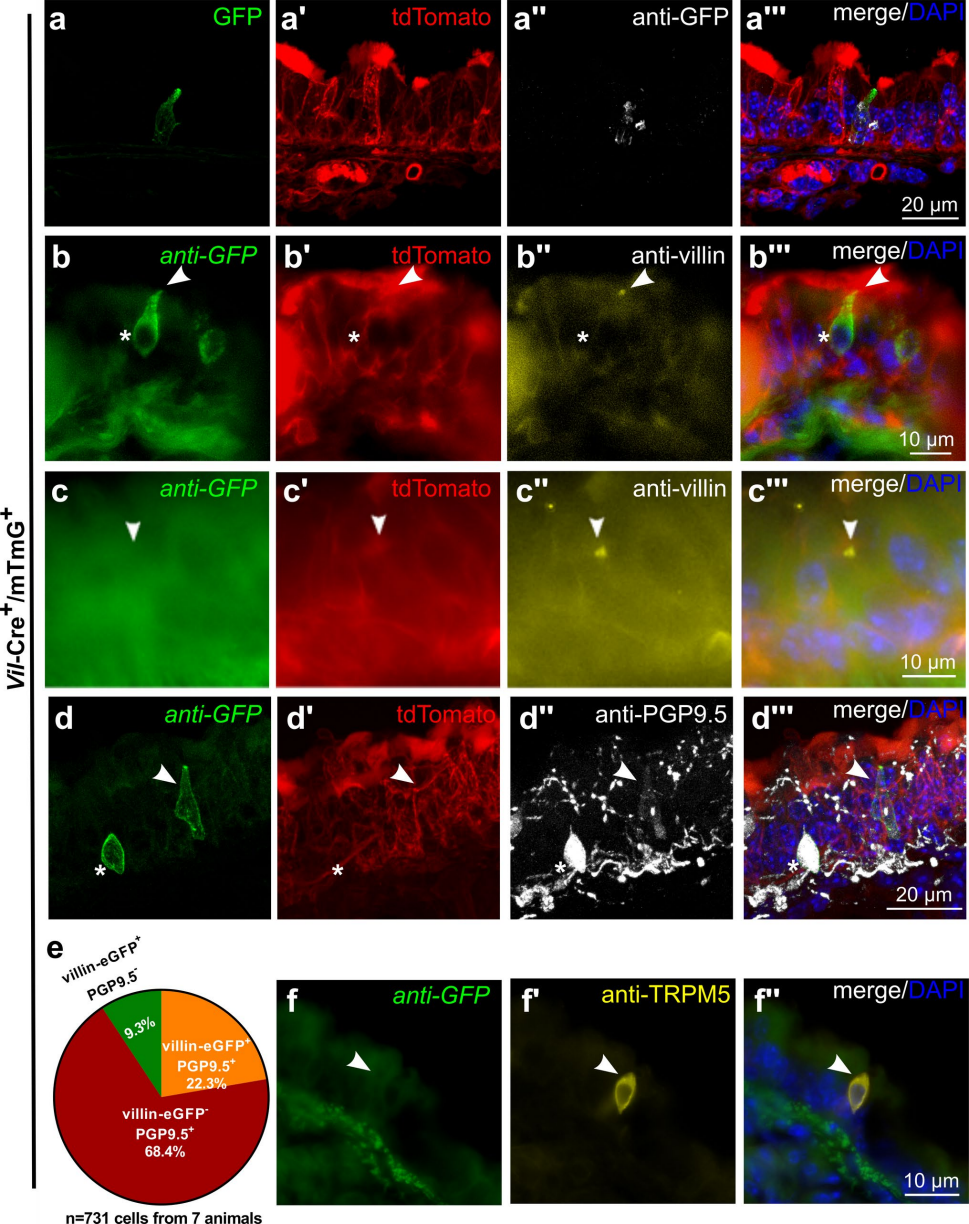
A subpopulation of NEC possesses *Vil1-Cre* activity. **a**–**d**, **f** Tracheal cryosections of *Vil1-Cre*^+^*/mTmG*^+^ mice. **a** Immunolabeling with antibodies against GFP, showing a single epithelial cell with endogenous GFP signal (a) and labeled with antibodies against GFP (a’’). **b**, **c** Immunolabeling with antibodies against GFP and villin (rabbit, monoclonal, RRID AB_2537927). **b** Cell double positive for GFP (asterisk) and villin (arrowhead). **c** Cell only positive for villin (arrowhead). **d** Immunolabeling with antibodies against GFP and PGP9.5 (rabbit, polyclonal, RRID AB_1952407), cell double positive for GFP and PGP9.5 (asterisk) and single positive only for GFP (arrowhead). **e** Pie chart showing the percentage of GFP^+^/PGP9.5^+^, GFP^+^/PGP9.5^−^, and GFP^−^/PGP9.5.^+^ cells related to the pooled total number of immunoreactive cells (6–8 coronal sections/trachea, *n* = 7 tracheas). **f** Immunolabeling with antibodies against GFP and TRPM5

In tracheal cryosections from *Vil1-Cre*^+^*/mTmG*^+^ animals, native eGFP was detected in single rare pyramidal or flask-shaped cells but not outside the epithelium (Fig. 7a). When sections were immunolabeled with an antibody against GFP, all native eGFP^+^ cells were also immunoreactive, and no additional cells became visible (Fig. 7a). Double-immunolabeling with antibodies against eGFP and villin revealed that Cre-recombinase was active in a portion of, but not in all, villin-immunoreactive epithelial cells (Fig. 7b, c). Double-labeling with antibodies against GFP and PGP9.5 (NEC-specific marker) revealed *Vil1*-driven eGFP in about one-quarter of PGP9.5-immunoreactive NEC (Fig. 7d, e). Double-immunolabeling with antibodies against GFP and the TC-marker TRPM5 did not reveal *Vil1-Cre* activity in TC (Fig. 7f).

**Fig. 8:**
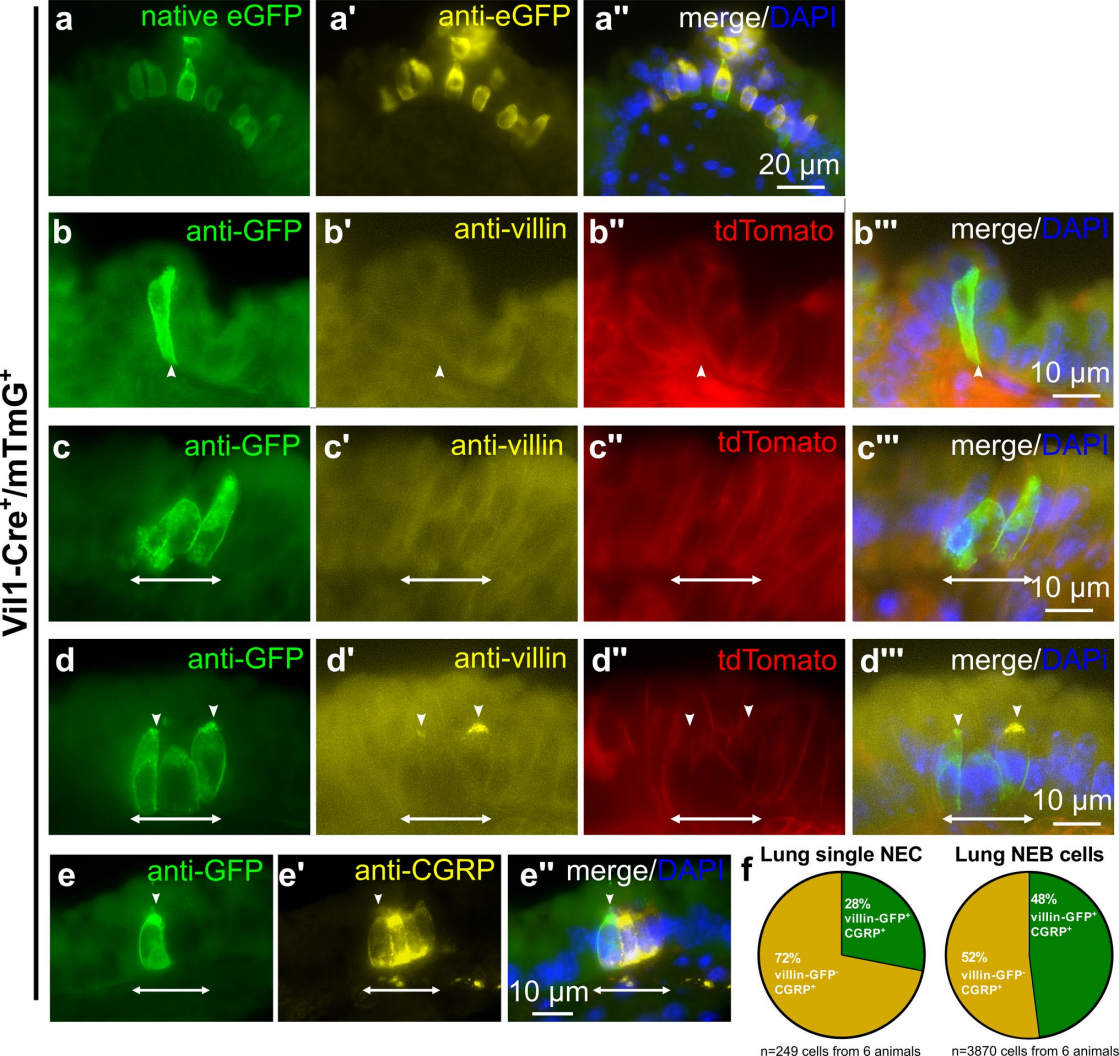
A subpopulation of pulmonary solitary NEC and NEB cells possess *Vil1-Cre* activity. **a**–**e** Lung cryosections of *Vil1-Cre*^+^*/mTmG*^+^ mice. **a** Immunolabeling with antibodies against GFP, showing a cluster of epithelial cells with endogenous GFP signal which are additionally labeled with antibodies against GFP. **b**–**d** Immunolabeling with antibodies against GFP and villin (rabbit, monoclonal, RRID AB_2537927), showing single and clustered eGFP-positive cells which are not labeled with antibodies against villin (**b** and **c**) and a cluster of double-positive cells (**d**, arrowheads); double-headed arrow indicates dimensions of the NEB. **e** Immunolabeling with antibodies against GFP and CGRP (rabbit, monoclonal, RRID AB_518147), showing a NEB with CGRP-immunoreactivity and a single cell within this cluster with additional GFP labeling (arrowhead); double-headed arrow indicates dimensions of the NEB. **f** Pie chart showing the percentages of GFP^+^/CGRP^+^ and GFP^−^/CGRP^+^ single cells (NEC; *n* = 249 cells from four animals) or cells within a cluster (NEB; *n* = 3870 cells from four animals), relative to the pooled total number of immunoreactive cells

In lung cryosections from *Vil1-Cre*^+^*/mTmG*^+^ animals, native eGFP fluorescence was detected in single flask-shaped and clustered flask-shaped or rounded cells (Fig. 8a, b). When such sections were immunolabeled with antibodies against GFP, all native eGFP^+^ cells were also immunoreactive, and no additional cells became visible (Fig. 8a). In *Vil1-Cre*^+^*/mTmG*^+^ animals, double-immunolabeling with antibodies against GFP and villin revealed single and clustered GFP-immunoreactive cells, but most of them were not villin-immunoreactive (Fig. 8b, c), and double-labeled cells were rare (Fig. 8d). Double-immunolabeling with antibodies against CGRP and GFP showed *Vil1*-driven GFP in less than one-third of solitary CGRP^+^ neuroendocrine cells, but in nearly half of the CGRP^+^ cells arranged in neuroepithelial bodies (Fig. 8e, f).

### Tracheal NEC express Vil1-mRNA

**Fig. 9:**
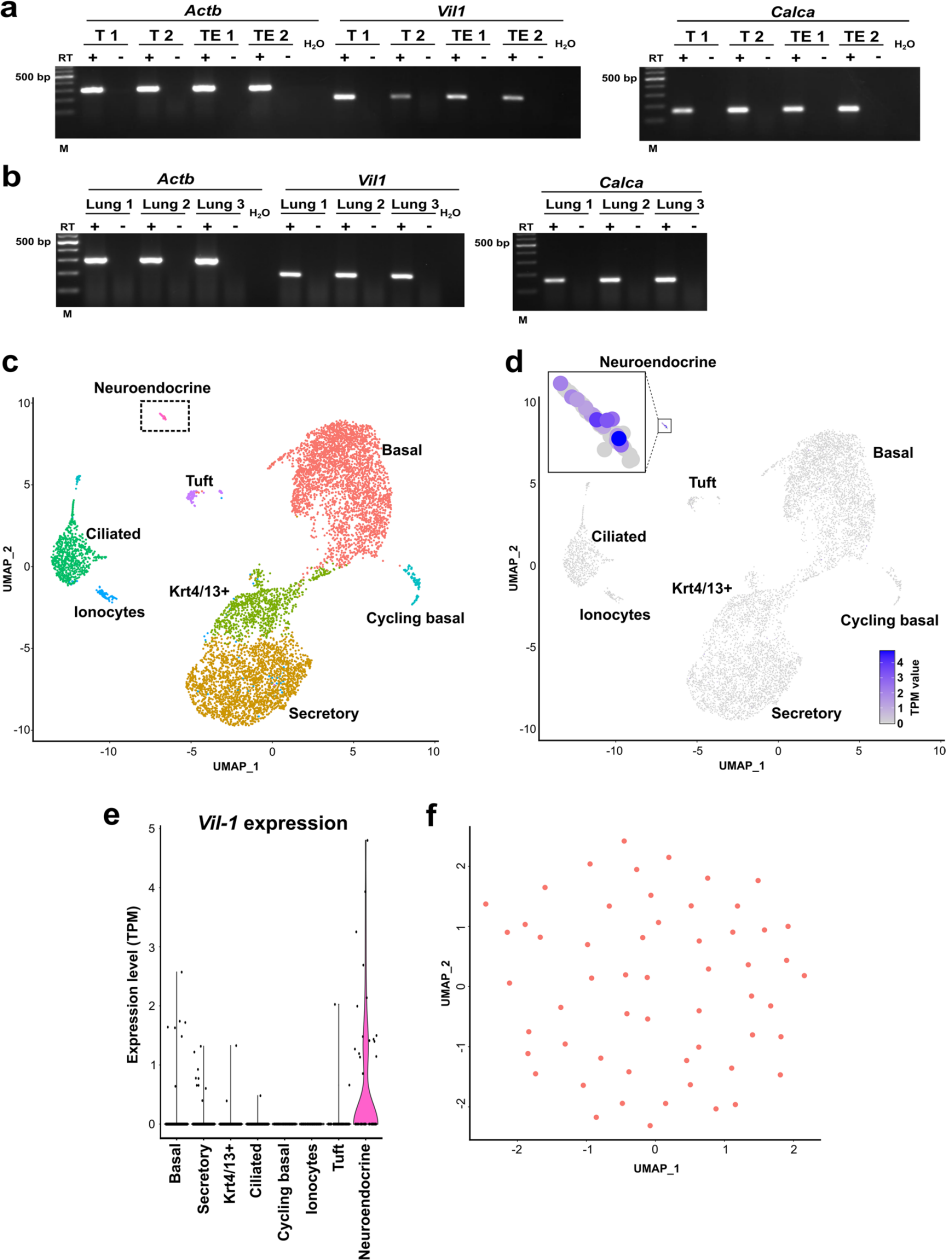
Vil1-mRNA is predominantly expressed by neuroendocrine cells in the tracheal epithelium. **a** RT-PCR of tracheas (T) and tracheal epithelium (TE) of two C57BL/6RJ mice each. **b** RT-PCR of three lungs of C57BL/6RJ mice. Amplicons of β-actin (*Actb*, 300 bp), Villin-mRNA (V*il-1*) (195 bp) and *Calca* (158 bp) were detected in all investigated samples. Controls = RNA samples processed without reverse transcriptase (RT-) and water (H2O) without adding cDNA. **c**–**f** In silico analysis of published sequencing data (GSE102580) of murine tracheal epithelial cells (Plasschaert et al. 2018). **c** SPRING plot (Uniform Manifold Approximation and Projection, UMAP) shows eight distinct cell clusters, namely basal, secretory, Krt4/13 +, ciliated, cycling basal, ionocytes, ciliated, tuft cells, and neuroendocrine cells. **d** SPRING plot showing that V*il-1* is predominantly expressed within the neuroendocrine cell cluster (insert). **e** Violin plot showing that *Vil-1* is predominantly expressed within the neuroendocrine cell cluster. **f** Unbiased analysis of the neuroendocrine cell cluster did not indicate different neuroendocrine cell subtypes based on distinctive gene expression patterns

Whole tracheas (*n* = 2), mechanically abraded tracheal epithelia (*n* = 2), and lungs (*n* = 3) of C57BL/6Rj mice were analyzed by RT-PCR utilizing primers specific for *villin (Vil1)* and *Calca*, the CGRP encoding gene. These experiments revealed the expression of mRNA of both targets in all investigated samples (Fig. 9a, b). At single-cell level, expression was analyzed in silico utilizing published sequencing data (GSE102580) of murine tracheal epithelial cells (Plasschaert et al. 2018). We were able to reproduce the clustering reported by Plasschaert and coworkers and identified eight distinct cell clusters, namely basal, secretory, Krt4/13^+^ (Keratin 4/13), ciliated, cycling basal, ionocytes, TC, and NEC (Fig. 9c). *Vil1*-mRNA was expressed predominantly in NEC, while its expression was negligible in other epithelial cell types. In this data set, less than one-third of NEC were expressing *Vil1-*mRNA (15/53 cells) (Fig. 9d, e). Unbiased analysis, however, allowed no subclustering of NEC based on distinct gene expression patterns, indicating that villin^+^- and villin^−^-NEC do not represent distinct NEC subtypes (Fig. 9f).

### The majority of TC is advillin-immunoreactive

**Fig. 10:**
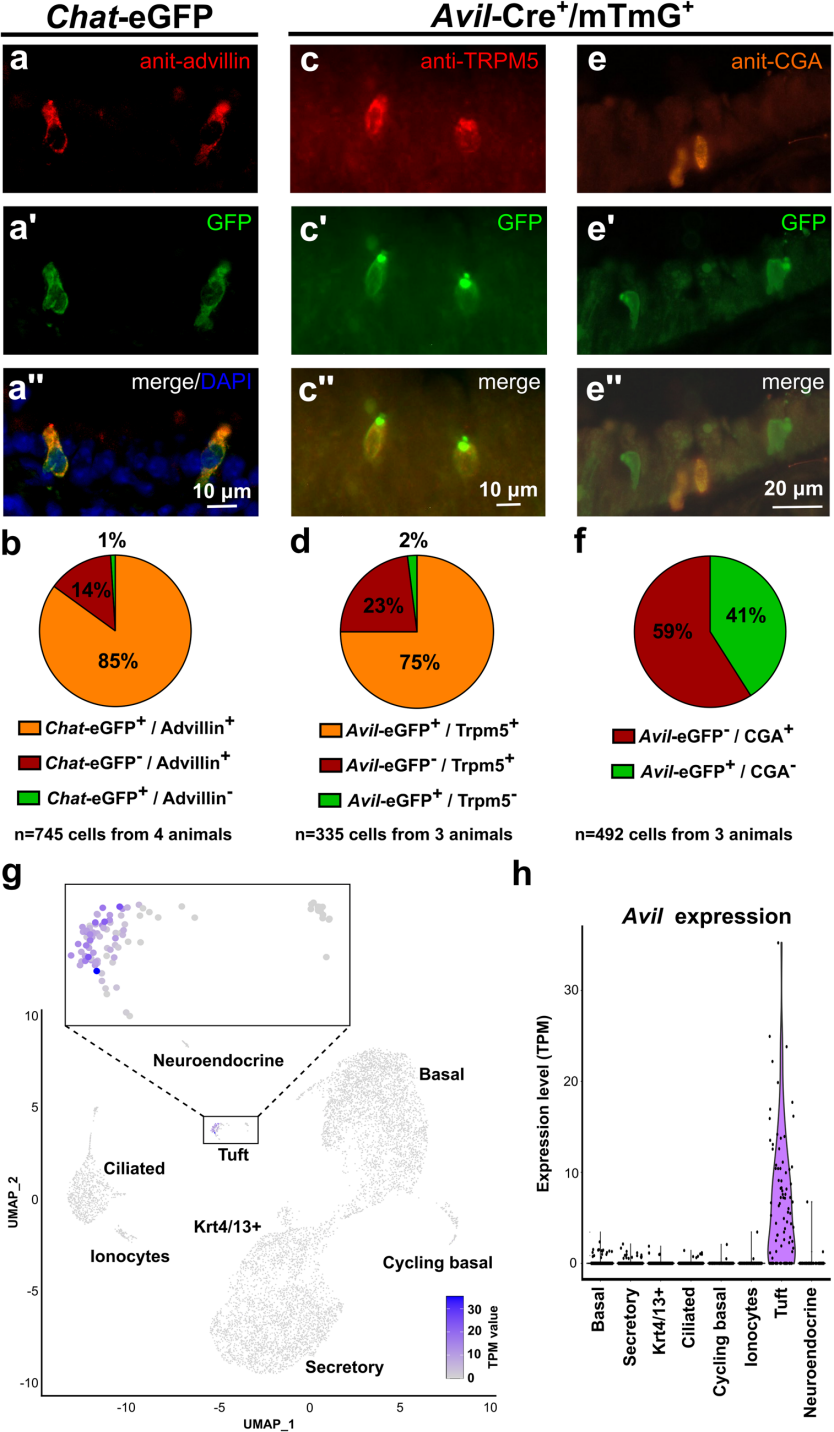
Advillin is expressed by tuft cells. **a** Immunolabeling of tracheal cryosections of *Chat*-eGFP mice, showing single epithelial cells with native GFP signal (a’), additionally labeled with antibodies against advillin (a). **b** Pie chart showing the percentage of eGFP^+^/Advillin^+^, eGFP^−^/Advillin^+^, and eGFP^+^/Advillin^−^ cells related to the pooled total number of immunoreactive cells (745 cells from four tracheas). **c** Double-immunolabeling of tracheal cryosections of Avil-Cre^+^/mTmG^+^ mice with antibodies against GFP and Trpm5, showing two single epithelial cells with co-labeling. **d** Pie chart showing the percentage of eGFP^+^/TRPM5^+^, eGFP^−^/TRPM5^+^, and eGFP^+^/TRPM5^−^ cells related to the pooled total number of immunoreactive cells (335 cells from three tracheas). **e** Double-immunolabeling of tracheal paraffin sections of Avil-Cre^+^/mTmG^+^ mice with antibodies against GFP and Chromogranin A (CGA, neuroendocrine cell marker). Note the lack of co-labeling. **f** Pie chart showing the percentage of eGFP^−^/CGA^+^ and eGFP^+^/CGA^−^ cells related to the pooled total number of immunoreactive cells (492 cells from three tracheas). **g**, **h** In silico analysis of the same data set analyzed in Fig. 9. **g** SPRING plot showing that *Avil-*mRNA is predominantly expressed within the tuft cell cluster (insert). **h** Violin plot showing that *Avil*-mRNA is predominantly expressed within the tuft cell cluster

Tracheal cryosections from *Chat*-eGFP reporter mice (*n* = 4) were immunolabeled with antibodies against GFP (to enhance fluorescence of *Chat*-eGFP^+^ cells; TC) and advillin. Rare polarized, flask-shaped epithelial cells were advillin-immunoreactive; labeling was distributed throughout the cell. GFP and advillin-immunoreactivities were co-localized in the majority of labeled cells (85%) (Fig. 10a, b).

### The Avil promoter is active in TC

Tracheal paraffin sections from Av*il-Cre*^+^*/mTmG*^+^ mice were double-immunolabeled with antibodies against GFP (to enhance fluorescence of advillin-eGFP^+^ cells) and TRPM5 (TC-specific marker). GFP-immunoreactivity, indicating *Avil-Cre* activity, was detected in three-quarters of TRPM5^+^cells (Fig. 10c, d). In contrast, double-immunolabeling with antibodies against GFP and chromogranin A (NEC-specific marker, Montoro et al. 2018) revealed no *Avil-Cre* activity in NEC (Fig. 10f).

### In silico analysis reveals Avil expression in tracheal TC

In silico analysis of the data set GSE102580 revealed *Avil*-mRNA expression predominantly in TC, while it was negligible in other epithelial cell types. In this data set, three-quarters of TC were expressing A*vi1-*mRNA (64/90 cells) (Fig. 10g, h).

## Discussion

Previous studies revealed the existence of a rare villin-positive cell in the mouse trachea that shared neither molecular markers nor developmental dependence on the transcription factor Pou2f3 with tuft cells, for which villin has commonly been considered a structural marker protein. The present study identifies this cell as a NEC rather than representing a novel entity beyond the known rare epithelial cell types, i.e., tuft cells, ionocytes, and NEC. Notably, villin is not ubiquitously expressed by airway NEC, ranging from occurrence in about two thirds of tracheal NEC to only a few in the lung when judged by protein detectability with antibodies. This heterogeneity indicates the existence of distinct subpopulations, which is in line with recent transcriptional and functional studies (Mahmoud et al. 2022; Schappe et al. 2024; Seeholzer and Julius 2024). In contrast to broncho-pulmonary NEC, tracheal NEC present high levels of the B-cell attracting chemokine CXCL13 (Mahmoud et al. 2022) and respond to aspiration-related chemical stimuli (acid, water) with release of ATP, which then excites nearby sensory neurons that initiate swallowing and expiration reflexes (Seeholzer and Julius 2024). In general, the receptors of such chemosensory cells are located at the apical microvilli protruding into the lumen and exhibiting a highly cell type-specific cytoskeletal architecture. The actin-bundling protein villin can be part of this complex machinery, as is the case in taste cells (Hofer and Drenckhahn 1999), but neither is it required to generate a microvillus nor is it expressed in all chemosensory cell types (Hofer and Drenckhahn 1999). In tracheal NEC, the presence of apical microvilli has been noted in a short review description (Kummer et al. 2008), but their molecular architecture has not been directly addressed yet. We here show that villin is one of the structural components of these specialized sensory microvilli. It remains to be determined whether tracheal NEC without detectable villin-immunoreactivity represent a truly distinct subpopulation or whether this observation is due to other causes such as sectional planes not containing the apical microvilli, imaging of immature cells before developing microvilli, or different functional states of the cells.

On the other hand, distinct features of pulmonary NEC, organized in NEB, are the synthesis of serotonin, the expression of osteopontin and cocaine- and amphetamine-regulated transcript, and mechanosensitivity, conveyed by the mechanosensory ion channel PIEZO2 (Piezo-type mechanosensitive ion channel component 2) (Schappe et al. 2024; Seeholzer and Julius 2024). They detect thorax compression and airway closure and evoke a gasp or sigh through a reflex mediated by the vagus nerve (Schappe et al. 2024). Mechanoreception does not require contact to the lumen. Accordingly, in mice, most of the surface of NEB is covered by specialized club cells, and only a few NEC have their apical surface exposed to the bronchiolar lumen and bear microvilli (Hung et al. 1979; Hung and Loosli 1974; Wasano and Yamamoto 1981). An abortive program to generate microvilli in cells which finally did not succeed in reaching the lumen and growing microvilli may underlie the numerous (nearly 50%) NEB cells having a history of *Vil* promoter activity without detectable villin-immunoreactivity at the time point of investigation. Shortly before birth, the entire luminal surface area is covered by microvilli, but beginning early after birth and then progressing, cells appear with large central bare areas surrounded only by a rim of microvilli (Haller 1994). Thus, in the adult murine lung, NEBs and most NEC are devoid of microvilli at all, and there is rarefication of microvilli in the majority of those which principally can build up microvilli. This alone might suffice to explain our observation that only a minimal fraction of NEB cells exhibited villin-immunoreactivity, although a different molecular microvillus architecture might also add to this.

In our study, solitary bronchopulmonary NEC shared with clustered NEC (NEBs) the features of only exceptional villin-immunoreactivity and a higher degree of history in *Vil* promoter activity. Their functional properties are less investigated, but about 50% exhibit also mechanosensitivity (Seeholzer and Julius 2024). So far, their ultrastructure has not been reported, and it is unclear whether they reach the epithelial surface and project microvilli into the lumen. On the light microscopical level, their morphology has been described as rounded, in contrast to that of tracheal and laryngeal NEC extending snout-like luminal projections (Seeholzer and Julius 2024), which matches our observations. Hence, as for clustered NEC, the lack of villin-immunoreactivity might simply reflect the lack of a luminal contact area with microvilli, but further ultrastructural studies are needed to substantiate this assumption.

While villin antibodies have been used historically to label TC (Hofer and Drenckhahn 1992, 1996; Krasteva et al. 2011; Krasteva-Christ et al. 2015; Yamashita et al. 2017), our findings suggest that advillin, rather than villin, is a more specific marker for TC in the murine trachea and other organs (Bezencon et al. 2008; Esmaeilniakooshkghazi et al. 2020, Ruppert er al., 2020). Furthermore, cell-type-specific deletion of ChAT in trachea and gallbladder TC has been successfully achieved using *Avil-Cre* mice to generate *Avil*^*cre*^*:ChAT*^*fl/fl*^ mice (Keshavarz et al. 2022; Perniss et al. 2023, 2020)*,* further confirming advillin as a reliable TC marker in the trachea. The structural similarity between villin and advillin (Marks et al. 1998) raises the possibility of antibody cross-reactivity, potentially leading to inaccurate conclusions in earlier studies that used villin to identify TC. The potential misinterpretation of villin immunoreactivity raises doubts about the appropriate identification of tracheal TC and the selection of suitable markers and driver lines for genetic studies.

In summary, this study uncovers villin-expressing cells in the lower airways as a cell population hidden among NEC. Advillin, not villin, is a marker for airway TC. This shall be considered in interpreting findings based on the use of villin as a marker when investigating TC.

## Supplementary Information

Below is the link to the electronic supplementary material.

Supplementary Material 1 (PDF 989 KB)

## Data Availability

No datasets were generated or analysed during the current study.

## Abbreviations

Avil: Gene name of advillin
BC: Basal cell
Calca: Calcitonin-related polypeptide alpha
CGRP: Calcitonin gene-related peptide
CFTR: Cystic fibrosis transmembrane conductance regulator
ChAT: Choline acetyltransferase
CXCL13: C-X-C motif chemokine ligand 13
DAPI: 4′,6-Diamidino-2-phenylindole
DCV: Dense core vesicles
eGFP: Enhanced green fluorescent protein
EDTA: Ethylenediaminetetraacetic acid
GFP: Green fluorescent protein
GNAT3: G protein subunit alpha transducin 3
HEPES: 4-(2-Hydroxyethyl)-1-piperazineethanesulfonic acid
Krt: Keratin
mTmG: Membrane-targeted tandem Tomato/membrane-targeted green fluorescent protein
NEB: Neuroepithelial bodies
NEC: Neuroendocrine cell
PCA: Principle component analysis
PGP: Protein gene product
Piezo2: Piezo-type mechanosensitive ion channel component 2
Pou2f3: POU class 2 homeobox 3
PBS: Phosphate-buffered saline
RT-PCR: Reverse transcription polymerase chain reaction
RLT: RNA lysis buffer
SEM: Scanning electron microscope
scRNA-seq: Single-cell RNA sequencing
Svil: Supervillin
SPF: Specified pathogen free
TC: Tuft cells
TEM: Transmission electron microscope
tdTomato: Tandem-dimer tomato
TRPM5: Transient receptor potential cation channel subfamily M member 5
UCHL1: Ubiquitin carboxy-terminal hydrolase L
UMAP: Uniform manifold approximation and projection
Vil1: Gene villin 1
