# Supplementary material for: Differential expression of villin and advillin by neuroendocrine and tuft cells in the murine lower airways
Source: Cell Tissue Res. 2025 Sep 1;402(1):1–20. doi: 10.1007/s00441-025-04003-y (PMC12484096; doi:10.1007/s00441-025-04003-y)
Supplement: Supplementary file 1 — Supplementary Material 1 (PDF 989 KB) [file 441_2025_4003_MOESM1_ESM.pdf]

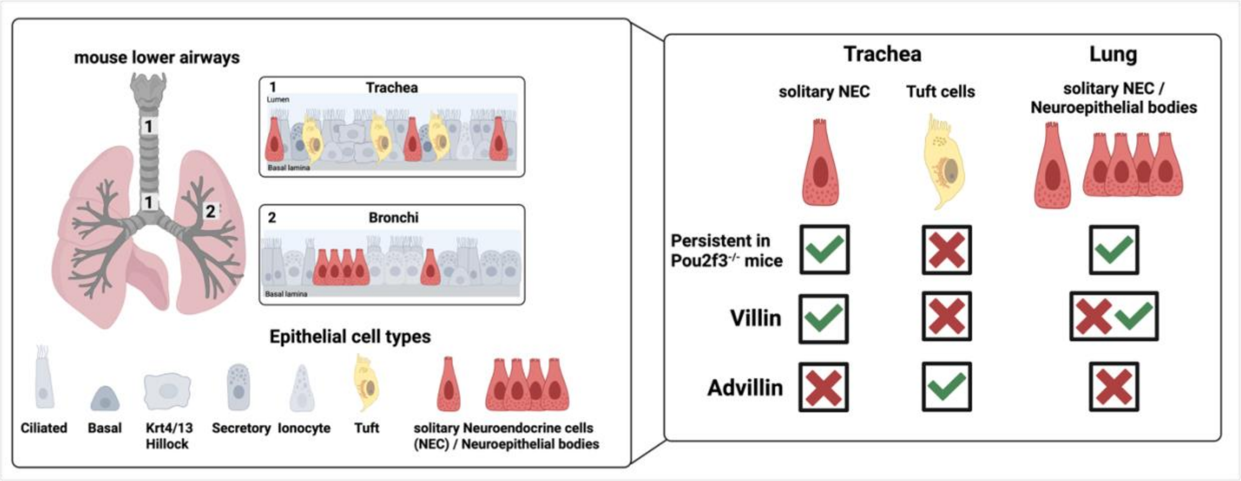

Graphical abstract: In mice, neuroendocrine cells express villin and persist in Pou2f3-deficient mice. Tuft cells, on the other hand, express advillin and are absent in Pou2f3-deficient mice.

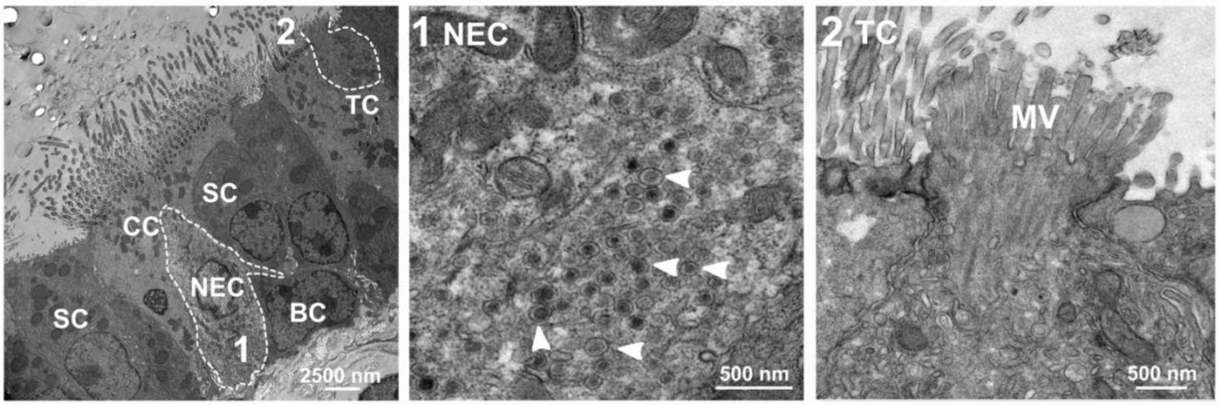

Supplementary Fig. 1: Electron microscopy of the tracheal epithelium of a C57BL/6Rj mouse, including CC, SC, BC, NEC, and TC. 1 is a higher magnification of the basal region of the NEC, showing the numerous DCV (arrowheads). 2 is a higher magnification of the apical region of the TC with MV. BC: basal cell; CC: ciliated cell; DVC: dense core vesicles; MV: microvilli; NEC: neuroendocrine cell; SC: secretory cell; TC: tuft cell.

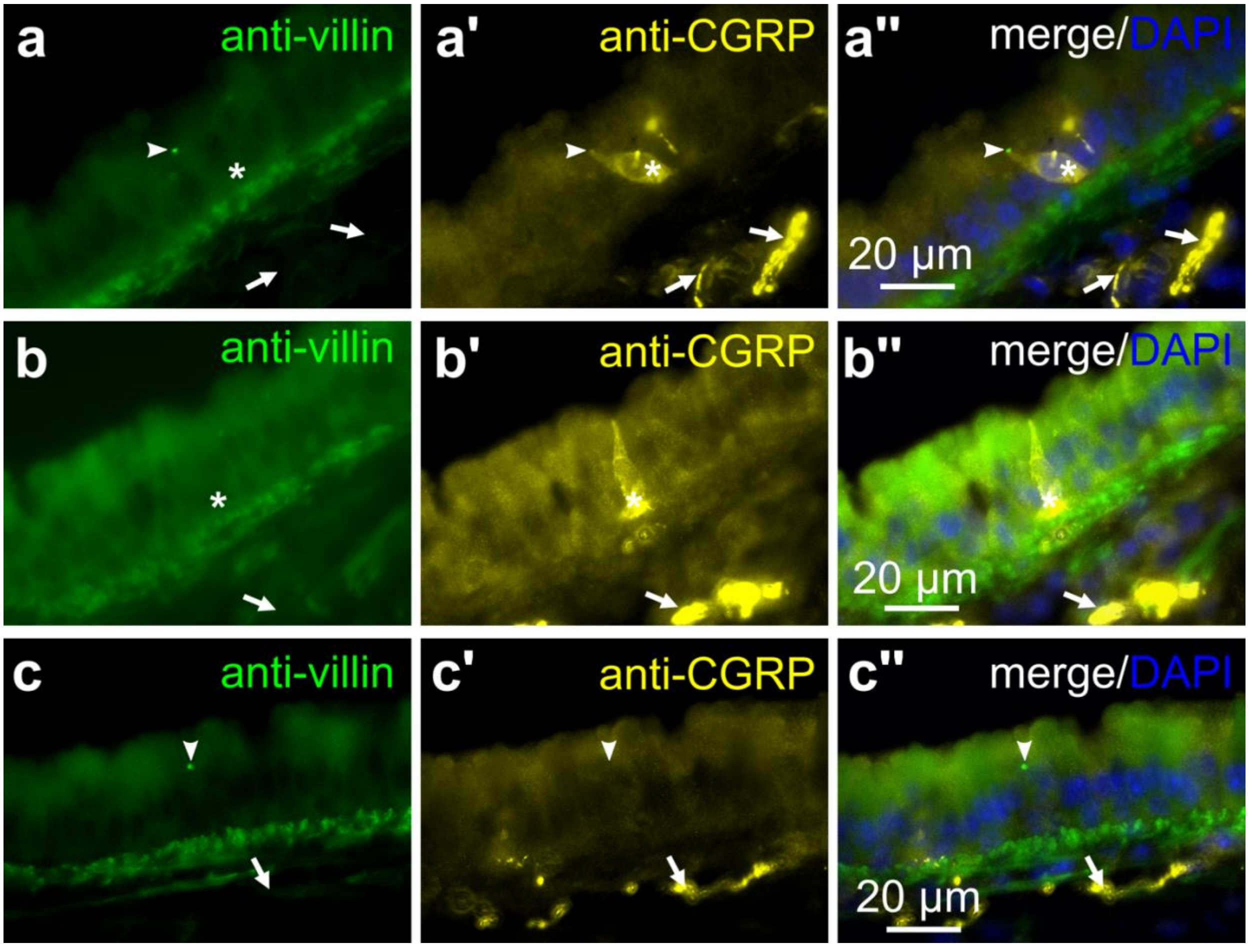

Supplementary Fig. 2: Villin- and CGRP-immunoreactivities in tracheal epithelium. (a-c) Immunohistochemistry of tracheal cryosections of C57BL/6RJ mice. Immunolabeled with antibodies against villin (rabbit, monoclonal, RRID AB_2537927) and CGRP (goat, polyclonal, RRID AB2243858). **(a)** Epithelial cell double-positive for villin (arrowhead, tip) and CGRP (asterisk). **(b)** Single CGRP^{+} cell (asterisk), not immunoreactive to villin antibody. **(c)** Single villin^{+} cell (arrowhead), not immunoreactive to CGRP antibody. **(a-c)** CGRP-immunoreactive nerve fibers are labeled by arrows.

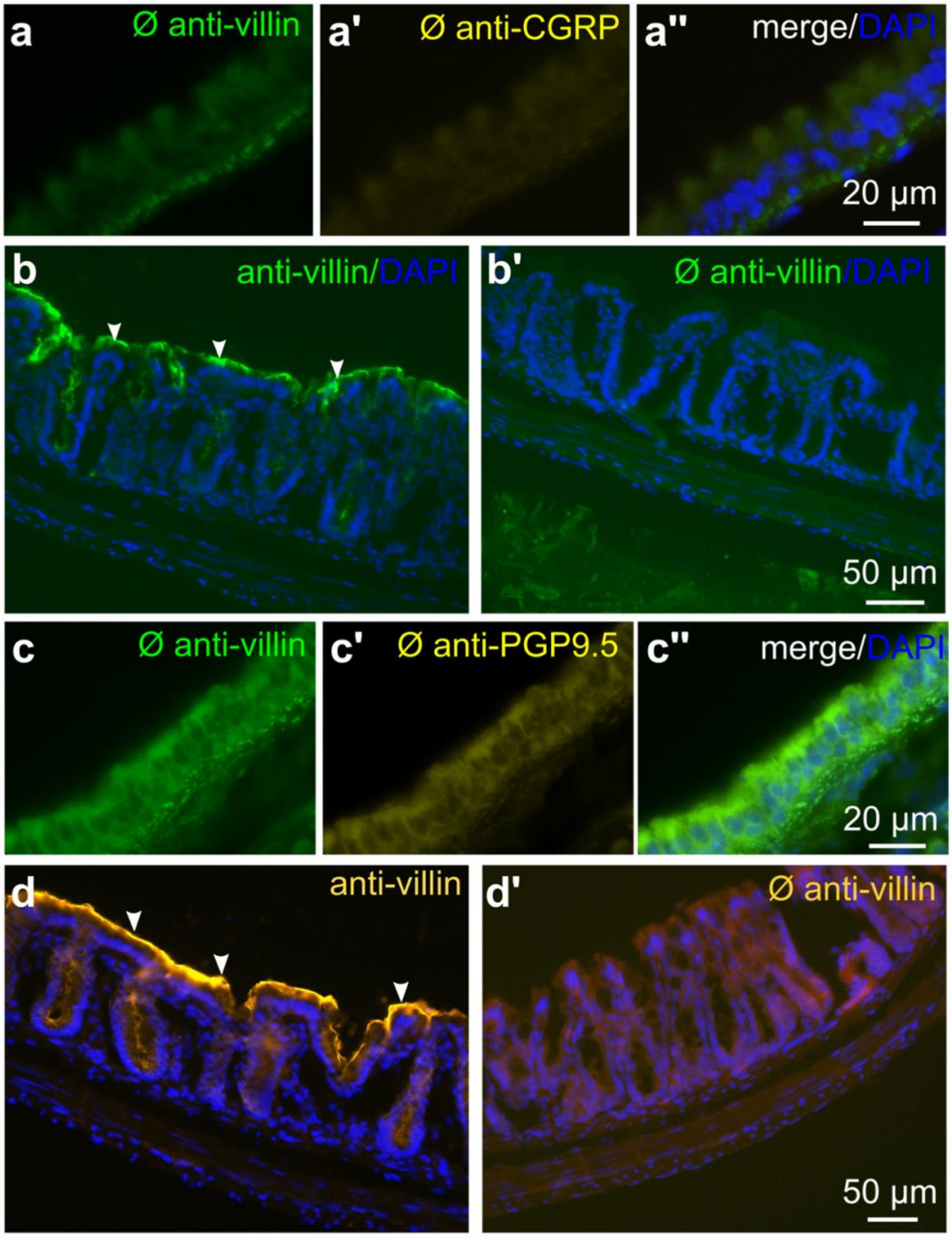

Supplementary Fig. 3: Controls for immunolabeling. (a) Immunohistochemistry of tracheal cryosections. No immunoreactivity is observed when all primary antibodies were omitted, same secondary antibodies as in Fig. S2 where used. **(b)** Immunohistochemistry of colon cryosections (C57BL/6RJ mice; positive control) immunolabeled with the same primary (rabbit, monoclonal, RRID AB_2537927) and secondary antibodies used for villin staining in this figure and Fig. 4 (a-c), showing villin-immunoreactivity of the brush border of colonocytes (arrowheads). **(b')** No immunoreactivity is observed when the primary antibody was omitted. Images captured with color camera; true colours are shown. (**c)** No immunoreactivity is observed when all primary antibodies were omitted, same secondary antibodies as in Fig. 4a-c were used. Images captured with colour camera; true colours are shown. **(d)** Immunohistochemistry of colon cryosections from (C57BL/6RJ) (positive control) immunolabeled with the same primary (rabbit, polyclonal, RRID AB_1968408) and secondary antibodies used for Fig. 4f, showing villin-immunoreactivity of the brush border of colonocytes (arrowheads). No immunoreactivity is observed when the primary antibody (villin) was omitted (d'), images captured with color camera; true colors are shown.

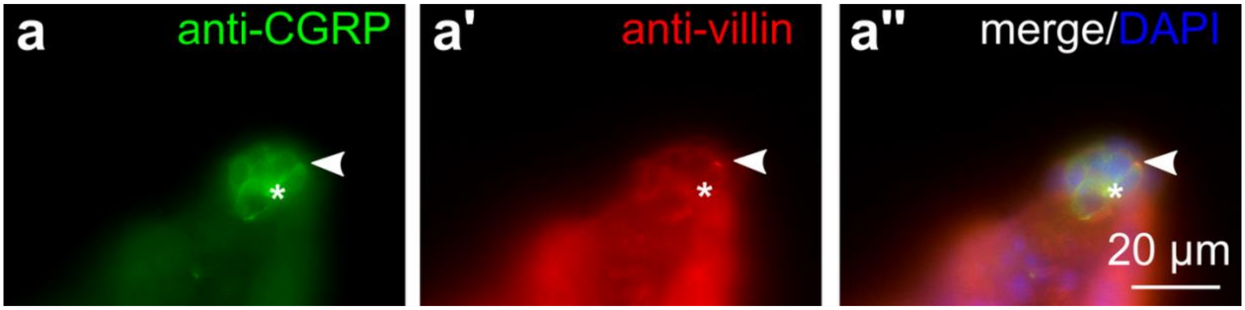

Supplementary Fig. 4: Villin- and CGRP-immunoreactivities in the intrapulmonary epithelium airway epithelium. Immunohistochemistry of lung cryosections of C57BL/6RJ mice. A single epithelial cell within a neuroepithelial body in the bronchial epithelium is double-positive for villin (rabbit, monoclonal, RRID AB_2537927, arrowhead, tip) and CGRP (goat, polyclonal, RRID AB2243858, asterisk), other $CGRP^+$ cells are $villin^-$.

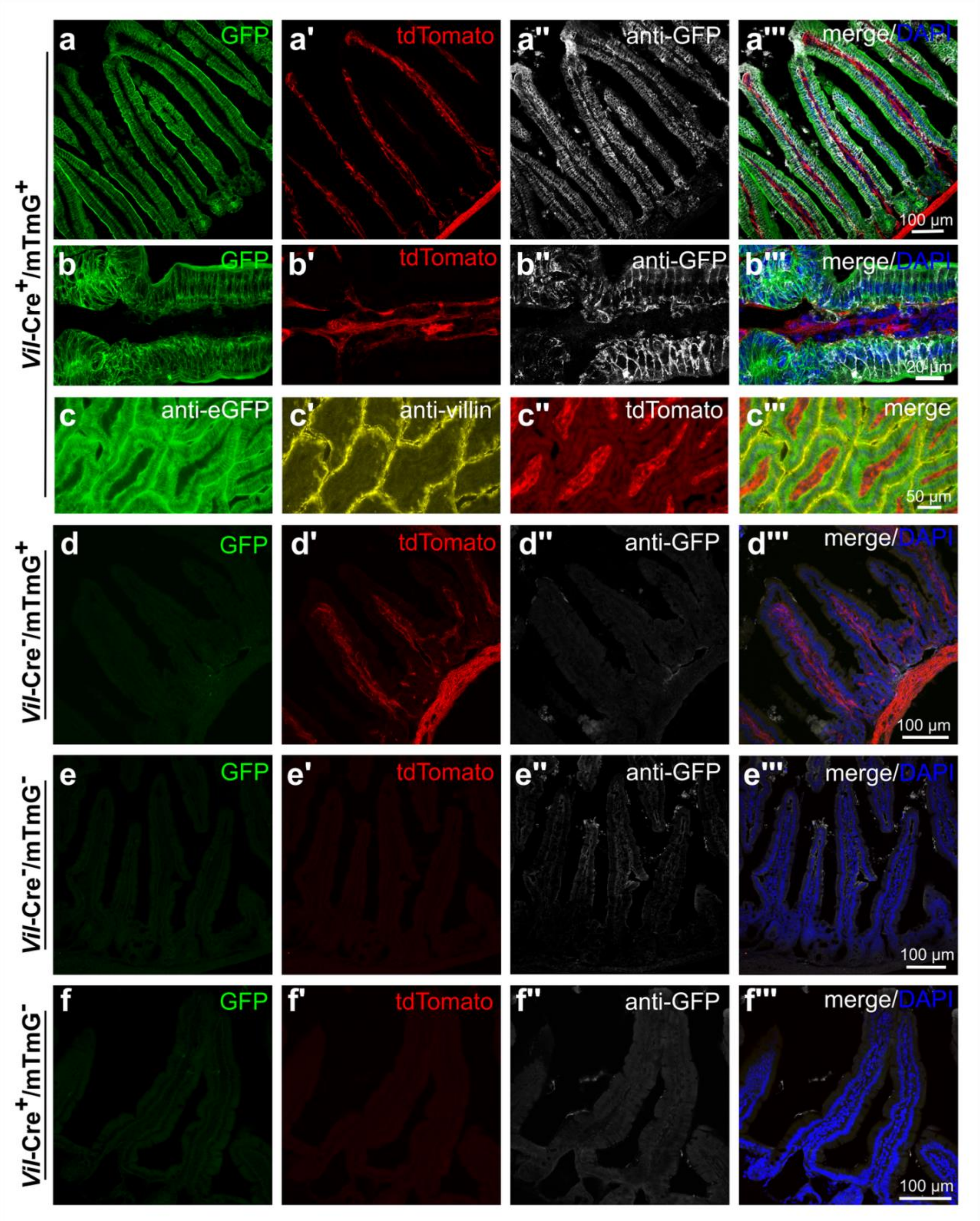

Supplementary Fig. 5: Validation of the *Vil1-Cre+/mTmG+* mouse model (small intestine). (a-c) Cryosections of the small intestine of *Vil1-Cre+/mTmG+* mice. **(a-b)** Native GFP fluorescence (a, b) and immunolabeling with antibodies against GFP (a'' and b''). **(c)** Immunolabeling with antibodies against GFP and villin (rabbit, monoclonal, RRID AB_2537927). **(d)** Cryosections of the small intestine of *Vil1-Cre-/mTmG+* mice, native GFP fluorescence (d), or immunolabeled with antibodies against GFP (d''). **(e)** Cryosections of the small intestine of *Vil1-Cre-/mTmG-* mice, native GFP fluorescence (e), or immunolabeled with antibodies against GFP (e''). **(f)** Cryosections of the small intestine of *Vil1-Cre+/mTmG-* mice, native GFP fluorescence (f), or immunolabeled with antibodies against GFP (f'').

Mouse strain	Official strain name	Source	Characteristics / use in manuscript	Breeding scheme
**C57BL/6Rj**	C57BL/6Rj	Janvier Labs	Wildtype mice	None
***Chat*-eGFP**	B6.Cg-Tg(RP23-268L19-EGFP)2Mik/J	Jackson Laboratory, JAX: 007902	Tuft cell identification - GFP expression under the control of the *Chat* promotor	Homozygous breeding
**Pou2f3$^{-/-}$**	Pou2f3^{tm1Abek}	Matsumoto et al., 2011	Tuft cell deficient mice	Homozygous breeding
Vil1-Cre	B6.Cg- Tg(Vil1-cre)997Gum/J	Jackson Laboratory, JAX: 004586	Cre-recombinase expression under the control of the *Vil1* promoter	see below for *Vil1-Cre/mTmG*
ROSA$^{mT/mG}$	B6.129(Cg)-Gt(ROSA)26Sortm4(ACTB−tdTomato,−EGFP)Luo/J	Jackson Laboratory, JAX: 007676	Double fluorescent reporter mouse line	See below for *Vil1-Cre/mTmG* and *Avil1-Cre/mTmG*
Vil1-Cre/mTmG	see above *Vil1-Cre* and *ROSA$^{mT/mG}$*	see above *Vil1-Cre* and *ROSA$^{mT/mG}$*	Double fluorescent reporter, cre recombinase expressing cells express GFP all other cells tdTomato	Hemizygous *Vil1-cre* mice were bred with homozygous *ROSA$^{mT/mG}$* mice
Avil-Cre	B6.Cg- Tg(Vil1-cre)997Gum/J	Jackson Laboratory, JAX: 004586	Cre-recombinase expression under the control of the *Avil* promoter	See below for *Avil-Cre/mTmG*
Avil-Cre/mTmG	see above *Avil-Cre* and *ROSA$^{mT/mG}$*	see above *Avil-Cre* and *ROSA$^{mT/mG}$*	Double fluorescent reporter, cre recombinase expressing cells express GFP all other cells tdTomato	Hemizygous *Avil-cre* mice were bred with homozygous *ROSA$^{mT/mG}$* mice

Supplementary Table 1: Used mouse strains.

Antigen	Host	Dilution	Source	RRID
**PGP9.5**	rabbit, polyclonal	1:4,000	GeneTex, Irvine, USA	AB_1952497
**UCHL1 (PGP9.5)**	chicken, polyclona	1:4,000	Novus Biologicals, Littleton, Colorado, USA	AB_877619
**αCGRP**	rabbit, polyclonal	1:20,000	Peninsula Laboratories, San Carlos, USA	AB_518147
**αCGRP**	goat, polyclonal	1:3,000	Biotrend, Cologne, Germany	AB_2243858
**GFP**	chicken, polyclonal	1:2,000-4,000	Novus Biologicals, Littleton, Colorado, USA	AB_10001164
**Villin**	rabbit, monoclonal	1:400	Invitrogen, Carlsbad, California, USA	AB_2537927
**Villin-1 (N-terminal)**	rabbit, polyclonal	1:400	Abgent, San Diego, California, USA	AB_1968408
**TPRM5**	rabbit, polyclonal	1:4,000	Kaske et al. 2007	AB_2315500
**Advillin**	rabbit, monoclonal	1:800	Novus Biologicals, Littleton, Colorado, USA	AB_3285581
**GNAT3**	goat, polyclonal	1:800	Covalab, Bron, France	Cat# Pab73402
**CXCL13**	goat, polyclonal	1:400-1:800	R&D Systems, Minneapolis, USA	AB_355378
**Chromogranin A**	rabbit, polyclonal	1:2,000	L. Eiden, NIH, Bethesda, USA (Schaefer et al. 1994)	Lenny10

Supplementary Table 2: Used primary antibodies.

Antigen	Host	Conjugate	Dilution	Source	Catalogue code
**rabbit Ig**	donkey	Cy3	1:2,000	Chemicon, Limburg, Germany	AP182C
**rabbit Ig**	donkey	Alexa 488	1:500	Invitrogen, California, Carlsbad, USA	A-21206
**rabbit Ig**	donkey	Cy5	1:400	Dianova, Hamburg, Germany	711-175-152
**chicken IgY**	donkey	FITC	1:400	Dianova	703-095-155
**chicken IgY**	donkey	Cy3	1:2,000	Dianova	703-166-155
**chicken IgY**	donkey	Cy5	1:400	Dianova	703-175-155
**goat Ig**	donkey	Alexa 488	1:1000	Molecular Probes, Eugene, Oregon, USA	A-11055
**goat Ig**	donkey	CF488A	1:1000	Biotium, Hayward, California, USA	B-20016-1
**goat Ig**	donkey	Cy3	1:1,600	Merck, Kenilworth, New Jersey, USA	AP180C

Supplementary Table 3: Used secondary antibodies.

Cell type	Mouse strain	Number of cells	Apical width (nm)	MV length (nm)	MV width (nm)	MV distance (nm)	Short axis mitochondrial length (nm)	Long axis mitochondrial length (nm)
Ciliated	C57BL/6Rj	28	5877	-	-	-	450	795
secretory	C57BL/6Rj	30	7697	-	-	-	591	965
TC	C57BL/6Rj	11	1438	614	103	25	278	544
NEC	C57BL/6Rj	5	1294	286	122	115	179	365
NEC	$Pou2f3^{-/-}$	15-16	1586	300	127	108	193	410

Supplementary Table 4: Ultrastructural features of tracheal epithelial cell types determined by TEM, MV = Microvilli.

Cell type	Mouse strain	Number of cells	Microvilli length (nm)	Microvilli width (nm)
TC	C57BL/6Rj	5	548	77
NEC	C57BL/6Rj	5	241	99

Supplementary Table 5: Identification of TC (Tuft cell) and NEC (Neuroendocrine cell) in a C57BL/6Rj mouse by SEM.
